# Genomic and cDNA selection-amplification identifies transcriptome-wide binding sites for the *Drosophila* protein sex-lethal

**DOI:** 10.1371/journal.pone.0250592

**Published:** 2021-05-24

**Authors:** Hiren Banerjee, Ravinder Singh

**Affiliations:** Department of Molecular, Cellular and Developmental Biology, University of Colorado at Boulder, Boulder, CO, United States of America; "INSERM", FRANCE

## Abstract

**Background:**

Downstream targets for a large number of RNA-binding proteins remain to be identified. The *Drosophila* master sex-switch protein Sex-lethal (SXL) is an RNA-binding protein that controls splicing, polyadenylation, or translation of certain mRNAs to mediate female-specific sexual differentiation. Whereas some targets of SXL are known, previous studies indicate that additional targets of SXL have escaped genetic screens.

**Methodology/Principal findings:**

Here, we have used an alternative molecular approach of GEnomic Selective Enrichment of Ligands by Exponential enrichment (GESELEX) using both the genomic DNA and cDNA pools from several *Drosophila* developmental stages to identify new potential targets of SXL. Our systematic analysis provides a comprehensive view of the *Drosophila* transcriptome for potential SXL-binding sites.

**Conclusion/Significance:**

We have successfully identified new SXL-binding sites in the *Drosophila* transcriptome. We discuss the significance of our analysis and that the newly identified binding sites and sequences could serve as a useful resource for the research community. This approach should also be applicable to other RNA-binding proteins for which downstream targets are unknown.

## Introduction

For several decades, genetic analyses and biochemical characterizations have offered complementary approaches to investigate developmental processes. Such studies have led to the identification of regulatory proteins in numerous cellular pathways. For a subset of these regulatory proteins, subsequent molecular genetic analysis and biochemical procedures have revealed downstream regulated genes. Nonetheless, while these approaches have hinted existence of additional downstream targets of numerous regulatory proteins, inherent limitations of these techniques have left additional putative targets to be identified. The approach of genomic SELEX is an attempt here to uncover potential new downstream targets which could serve as reagents for future detailed investigations into cellular and developmental processes.

In all organisms, sequence-specific RNA-binding proteins regulate gene expression post-transcriptionally at various levels. In *Drosophila melanogaster*, Sex lethal (SXL), which is an example of a sequence-specific RNA-binding protein [1, 2] acts as the master sex switch during somatic sexual differentiation by activating a hierarchy of alternative splicing events in females [*Sxl* -> *transformer (tra)* —> *doublesex (dsx)*] [3, 4].

SXL controls three important regulatory pathways—somatic sexual differentiation, dosage compensation, and the female germline stem cell differentiation. In XY somatic cells, the SXL protein is not synthesized due to an in-frame translation stop codon(s) in its male-specific exon. This results in failure to synthesize the TRA protein, leading to the synthesis of the fruitless (FRU) protein and of the male-specific isoform of *dsx* (DSX^**M**^) [5, 6]. In contrast, in XX flies, the SXL protein is synthesized due to alternative splicing. SXL causes synthesis of the TRA protein and thus production of the female-specific DSX^**F**^ isoform and absence of the FRU protein. Moreover, SXL prevents synthesis of the male-specific-lethal-2 (MSL2) protein to controls dosage compensation, which equalizes the expression of X-chromosome genes in both sexes.

At the molecular level, SXL regulates splice site choice in somatic cells by binding to specific uridine-rich RNA sequences and blocking adjacent splice sites in *Sxl*, *tra*, and *msl2* pre-mRNAs [7, 8]. It facilitates exon skipping in the *Sxl* pre-mRNA, 3’ splice site switching in the *tra* pre-mRNA, and intron retention in the *msl2* pre-mRNA. In addition, SXL represses the translation of its own mRNA and that of the *msl2* mRNA through SXL-binding sites in untranslated regions (UTRs) [9–12]. For poly(A) site switching, SXL competes with the binding of the polyadenylation factor CstF64 at the proximal polyadenylation signal, which in turn results in translation repression of the female germline-specific transcript specifically within the female germline [13].

Previous genetic studies suggested that SXL associates with numerous loci or nascent transcripts on polytene X-chromosomes in females [14] and likely regulates additional targets that remain to be identified [3, 4, 15]. Furthermore, SXL also regulates dosage compensation by an MSL2-independent mechanism(s) [16, 17]. SXL has been linked to transition from germline stem cells to committed daughter cells in ovaries and to ovulation [18, 19]. In the female germline, absence of SXL results in ovarian tumors [4, 15, 20]. In this regard, SXL has been shown to specifically target the *enhancer of rudimentary*, *Notch* signaling (*N* mRNA), and Antizyme/gutfeeling [13, 21, 22]. While SXL plays a central role in various aspects of sexual differentiation through these developmental or cellular processes, additional molecular targets remain to be identified.

Therefore, in our attempt to identify additional targets of SXL, we used Genomic Selective Enrichment of Ligands by Exponential enrichment (GESELEX) strategy. To accomplish this, we prepared the selection pool from the *Drosophila* genome as well as cDNA pools from developmental stages. Our unbiased and comprehensive analysis provides a transcriptome-wide view of potential SXL-binding sites in *Drosophila*.

## Results

We have used GESELEX to search for potential RNA targets on a transcriptome-wide basis. The GESELEX approach is a modification of the widely used technique of iterative selection-amplification or SELEX from a random library [23–25] or genomic library [26, 27]. In GESELEX, overlapping fragments (average 150 basepairs long) corresponding to the entire *Drosophila* genome or to the cDNA pools from different stages of development were attached to a T7 RNA polymerase promoter using a two-step random priming process with appropriate primers (for details see Materials and Methods). We emphasize that the pool from cDNA sequences should also contain potential SXL-binding sites because *Sxl*, *msl2*, *and enhancer of rudimenary* mRNAs, which are the known targets of SXL, contain SXL-binding sites in their untranslated sequences.

### Generation of genomic and cDNA libraries

**Fig 1:**
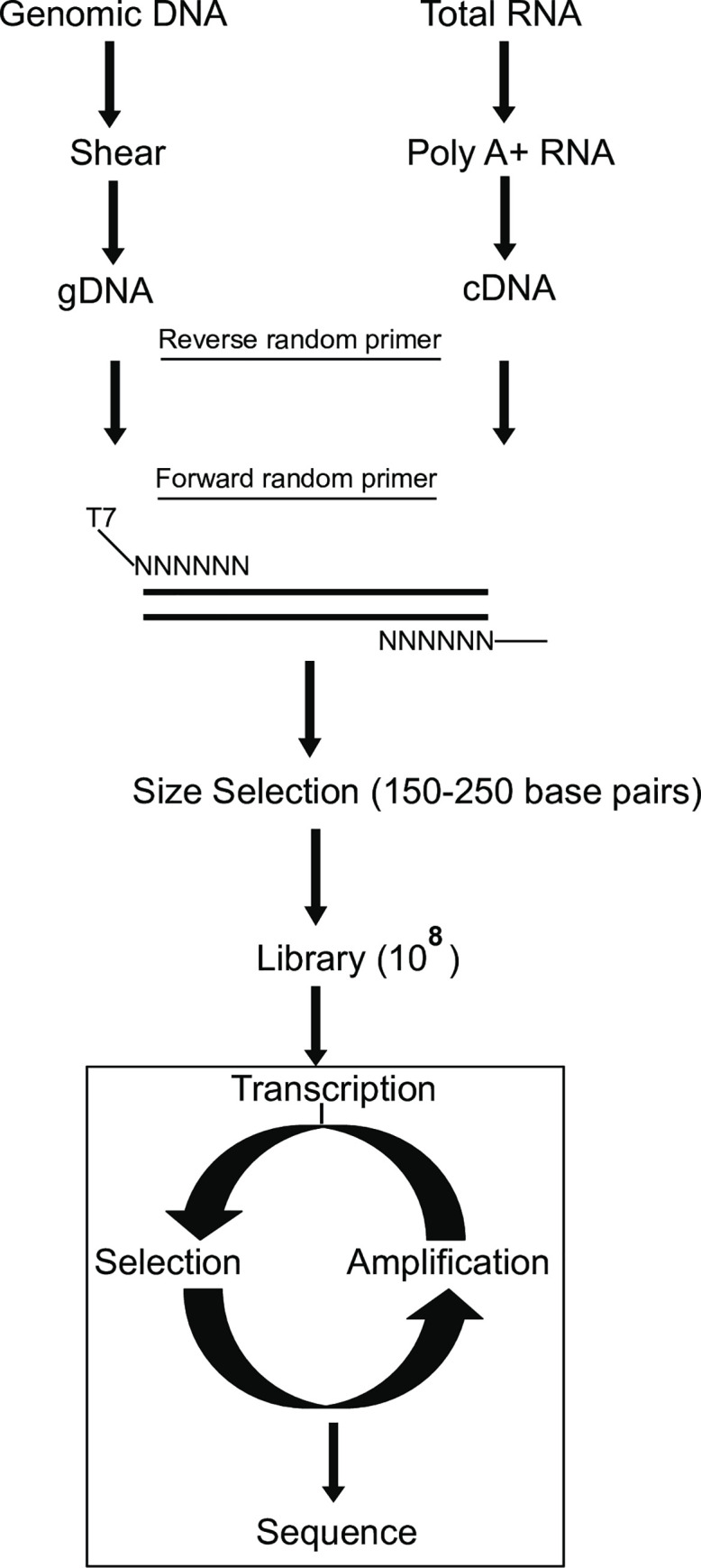
Schematics of the genomic and cDNA SELEX. Outline of steps to generate genomic and cDNA libraries. Process of genomic and cDNA SELEX (bottom).

A schematics for the generation of GESELEX libraries is shown in Fig 1. In brief, genomic DNA was isolated and sheared by passing through a fine-gauge needle. Then the first- and the second-strand synthesis was performed using primers with randomized sequences at their 3’ ends. The DNA sample was size fractionated on a polyacrylamide gel to obtain approximately 150 to 250 basepairs long product. During the process of DNA amplification, one of the primers attached T7 RNA polymerase promoter to the polymerase chain reaction (PCR) product. This PCR DNA provided a template for the synthesis of the pool 0 RNA corresponding to the *Drosophila* genome for genomic SELEX.

To obtain cDNA SELEX libraries from different developmental stages, total RNA was isolated from various developmental stages, for example, embryonic, larval, pupal, and adult flies. Poly(A)^**+**^ RNA fraction was obtained using oligo(dT) affinity selection. A reverse primer (SW3N7N9) with a randomized 3’ end was used for reverse transcription. The cDNA product was next subjected for second-strand synthesis using a forward primer (SW5N7N9) with a randomized 3’ end. The DNA fragments were size selected using polyacrylamide gel electrophoresis to obtain approximately 220 to 350 basepairs products. Alongwith another reverse primer (3N7.1) corresponding to the fixed sequence of the SW3N7N9 reverse primer, a second forward primer (5LN7.1) was used for PCR to add a T7 RNA polymerase promoter to the PCR product. These PCR products, or the cDNA libraries from various developmental stages, served as templates for synthesis of the pool 0 RNA corresponding to each of the *Drosophila* cDNA SELEX libraries.

**Fig 2:**
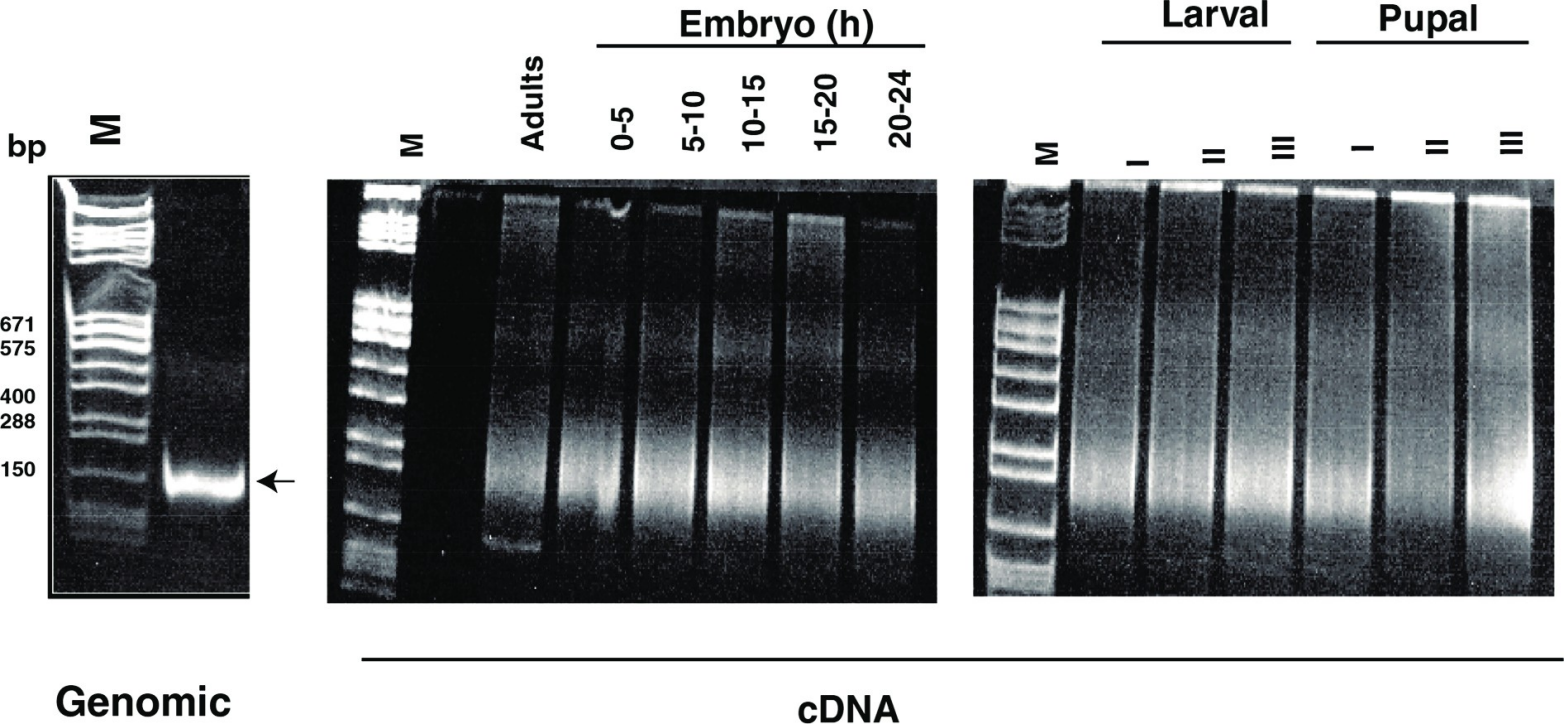
PCR products of the genomic and cDNA libraries. The source of DNA (*Drosophila* DNA) or mRNA from various developmental stages are shown at the top: embryonic 0–5 hrs, 5–10 hrs, 10–15 hrs, 15–20 hrs, and 20–24 hrs; larval I, II, and III instar stages; pupal I, II, and III instar stages; and adult flies. Arrow indicates the size (approximately 150 basepairs of the genomic library). Approximately 220–350 basepairs PCR products were excised for each of the cDNA libraries. Size markers are marked by the lanes labeled M.

Size-selected libraries (approximately 150–250 basepairs) using the genomic DNA and cDNA pools from different stages of development are shown in Fig 2. These include the genomic DNA library and the embryomic, larval, pupal, and adult cDNA libraries, as indicated at the top.

### Selection-amplification using genomic and cDNA libraries

Both the genomic and the cDNA SELEX libraries were subjected to iterative selection-amplification protocol, as described for the previous random SELEX protocol we have used [28]. In brief, the genomic DNA or cDNA libraries, were used for transcription *in vitro*. The RNA was gel purified and this pool 0 RNA from each of the pools was incubated with appropriate concentrations of the purified recombinant SXL protein. Following six rounds of selection-amplification with these libraries, we cloned and sequenced SXL-enriched sequences. We note that the protein-bound RNA fraction was obtained using a filter-binding assay for the initial rounds of selection-amplification and then by electrophoretic gel mobility shift assay for the last two rounds. The process of SELEX, which was similar for both the genomic and the cDNA libraries, is schematized in Fig 1.

As an evidence of successful selection-amplification, Table 1 shows representative sequences from the genomic pool. SXL-selected long uridine-rich sequences. For comparison, natural sequences from known SXL targets such as *Sxl*, *tra*, *msl2*, and *e(r)* are shown. Moreover, the consensus sequence for SXL-binding site is shown.

**Table 1:** Sequences of the SXL-binding sites from the genomic library.

Natural Targets of Sex-lethal	
tra	UUUUUGUUGUUUUUUUU
msl-2	UUUUUUUUUUUUUUUUG
Sxl-1	UUUUUUUUUUUCUUUUUU
Sxl-2	UUUUUUUUUUUGCAUAUUUUUUU
Sxl-3	UUUUUUUUUAUUUUUUUU
e(r)-1	UGUGUGUGUUUUUGUGUGUGUUUCAAUGUUUUUUUGUG
e(r)-2	GUAUGUUUGUUUGUUUG
e(r)-3	UUUUUUUUUGUAUGUUUUG
Random SELEX	
Consensus	UUUUUGUU[GU]U[GU]UUU[GU]UU
Genomic SELEX	
1	GUGUUUUUUUUUUGUUUUGUUUGUUUU
2	GUUUUUUUUUUUUUUUUUUUG
3	UUUUUUGUUUUUUUUUUUUUUUUUUG
4	UUUUUGUUUUUGUGUUGUUUUUUUU

For comparison, sequences of the natural binding sites for SXL are shown at the top, the consensus sequence, previously identified from random SELEX, is shown in the middle, and the genome-derived representative sequences are shown at the bottom.

Table 2 shows representative sequences from multiple cDNA pools: embryonic, larval, pupal, and adult. The SXL-selected sequences are long and uridine-rich and interupted by one or two guanosines or cytosines. We conclude that our genomic and cDNA selection-amplification approach has identifed on a transcriptome-wide level potential natural binding sites for the SXL protein.

**Table 2:** Sequences of the SXL- binding sites from the cDNA libraries.

Clone ID	SELEX sequences enriched
0-5h_1	UCCUGUUUUUGUUCUGUUUGU
5-10h_7	GUUUUUUUCUUUUUUUUUUUUUUUCC
10-15h_5	AUUUUUUUUUUUUCUUUUUUUCUAA
10-15h_14	GUUUUUUUUUUUUGUUUGUUGAC
15-20h_1	AUUUUUUUUUUGUUUUUCUUCGGGUUUU
20-24h_27	AUUUAUUUUGGUUUCAUUUUUUGUU
1 Larvae_41	GGUUUUUUUGUUUUUUUGUUAA
2 Larvae_12	GAAUUUUAUUCUUUUUUUUUUUUUA
3 Larvae_14	AUUUCUUUAAUUUUUUGUAUAUUUUU
1 Pupae_1	AGGUGUUCUUUUUAUUGCGUGGUACAGAUUUUAUUUU
2 Pupae_13	CAUUUUUUGUAUUAUUUUUGGUUUUAUUUUUUU
3 Pupae_37	AUUUUUCGUUUUUUUUUCUCUCCAAUUU
Adult_2	CAUUUUUUUUUUUUUUUUUCUCAUUUCC
Adult_16	GAAUUUUUAUUAUUUUUUUAUUAUUUG

Representative sequences derived from cDNA libraries from different developmental stages are shown.

While representative sequences from genomic and cDNA SELEX are shown in Tables 1 and 2, we have provided the remaining sequences from genomic and cDNA SELEX in S1 and S2 Tables. We have also provided additional information about gene names and chromosomal locations for the SXL-selected sequences from genomic and cDNA SELEX (S3 and S4 Tables).

### Binding affinities of SXL for the selected RNA pools

**Fig 3:**
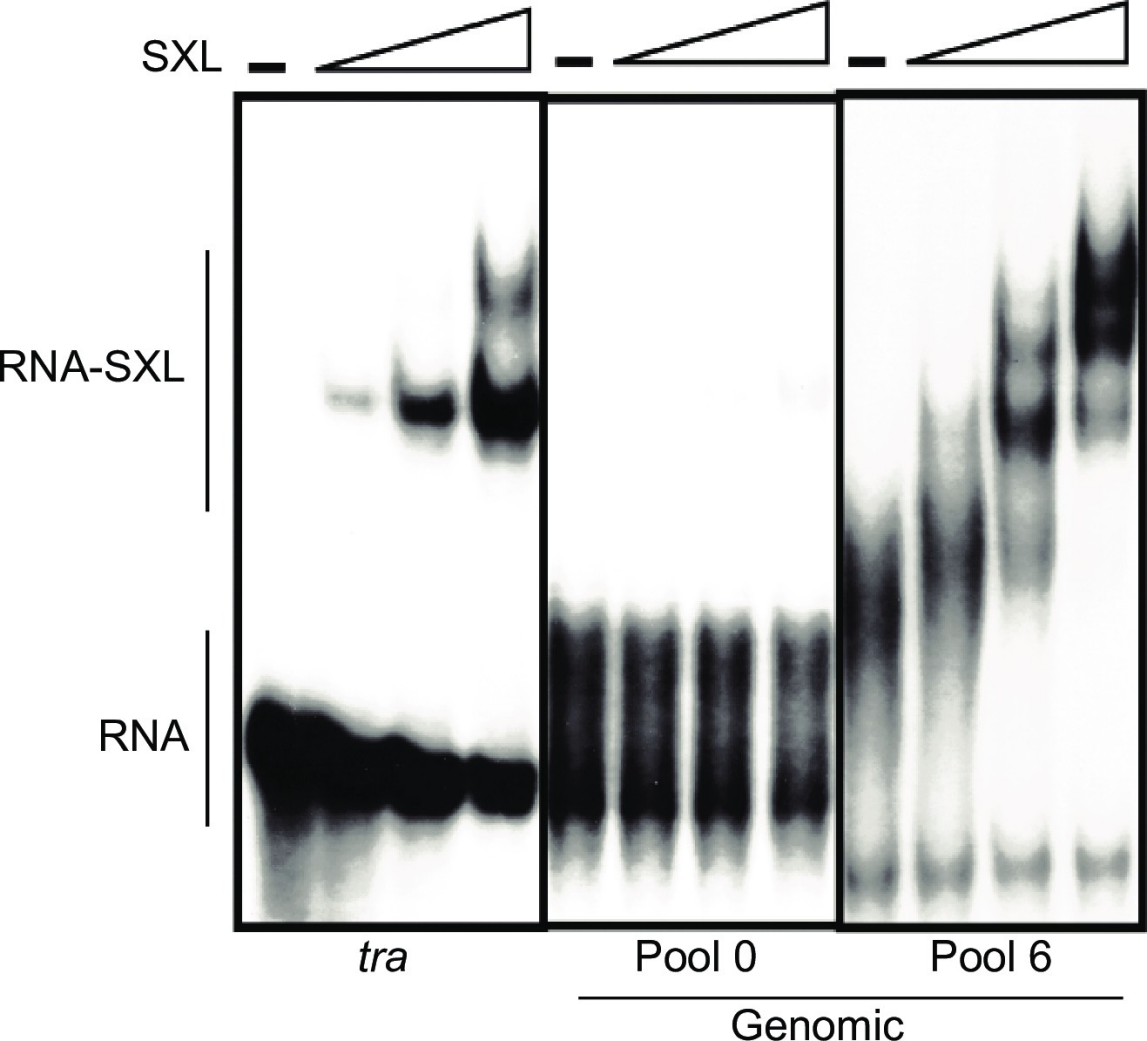
Binding of SXL to the RNA pools from the genomic SELEX library. Electrophoretic gel mobility assay was used to determine binding affinity of SXL with the non-sex-specific polypyrimidine-tract/3’splice site (a well characterized SXL binding site) of *tra*, the pool 0 RNA for the genomic library, and the pool 6 RNA of the SXL-selected sequences. No SXL is shown by the–lane, and increasing concentrations (three-fold successive increase) of the recombinant GST-SXL protein are shown by the triangles. The–lane shows the free (unbound) RNA, and RNA-protein complexes are shown under various SXL concentrations (166 ng/μl; 55 ng/μl; 18 ng/μl).

For biochemical analysis and characterization of the SXL-selected sequences, we examined binding affinities beween SXL and the selected sequences. SXL showed barely detectable binding for pool 0. However, SXL bound to the SXL-selected genomic pool (Pool 6) with high affinity. We note that the binding affinity for sequences in SXL-selected pool from the genomic DNA was comparable to that for one of the well-characterized SXL-binding site (non-sex-specific polypyrimidine tract) in the *tra* pre-mRNA (*K*_*d*_ *~* 1 nM) (Fig 3).

**Fig 4:**
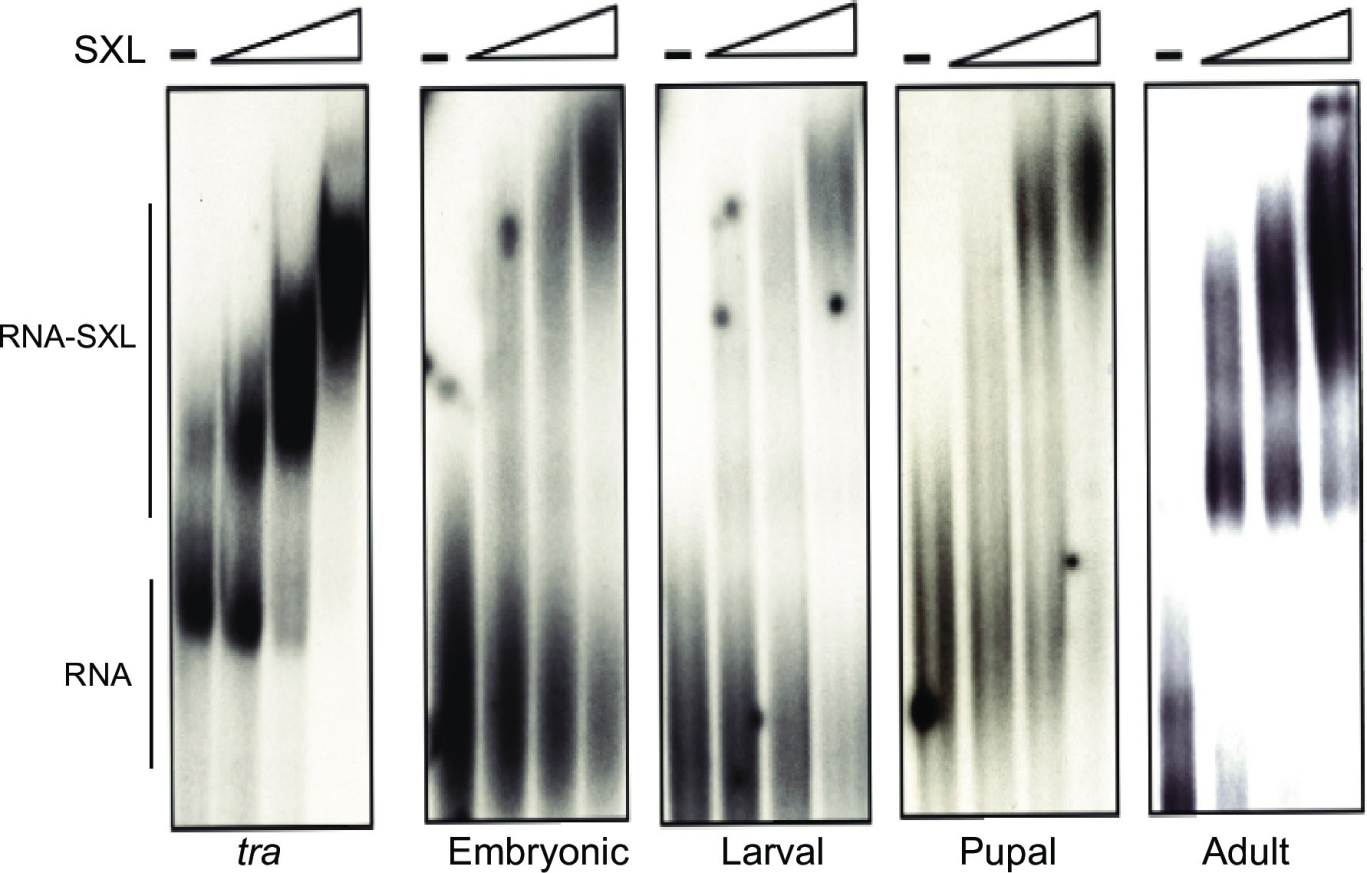
Binding of SXL to RNA pools from different cDNA SELEX libraries. Electrophoretic gel mobility assay was used to determine binding affinity of SXL with the non-sex-specific polypyrimidine-tract/3’splice site of *tra*, and the pool 6 RNA of the SXL-selected sequences for cDNA libraries (prepared from embryonic, larval, pupal, and adult polyA^**+**^ RNAs). No SXL is shown by the–lane, and increasing concentrations (three-fold successive increase) of the recombinant GST-SXL protein are shown by the triangles. The–lane shows the free (unbound) RNA, and RNA-protein complexes are shown under various SXL concentrations (166 ng/μl; 55 ng/μl; 18 ng/μl).

Furthermore, SXL bound to each of the SXL-selected cDNA pools (from embryonic, larval, pupal, and adult stages) with high affinity. We reiterate that binding affinities for sequences in SXL-selected pools for the cDNA libraries were also similar to that for the well-characterized SXL-binding site, the non-sex-specific polypyrimidine tract, in the *tra* pre-mRNA (our positive control) (Fig 4).

We conclude that SXL selected high-affinity sequences from both the genomic and the cDNA SELEX libraries.

### SXL-binds to uridine-rich sequences of *Troponin I* (*TnI)* and *Glutamine synthetase 2* (*Gs2*) transcripts with high affinity

**Fig 5:**
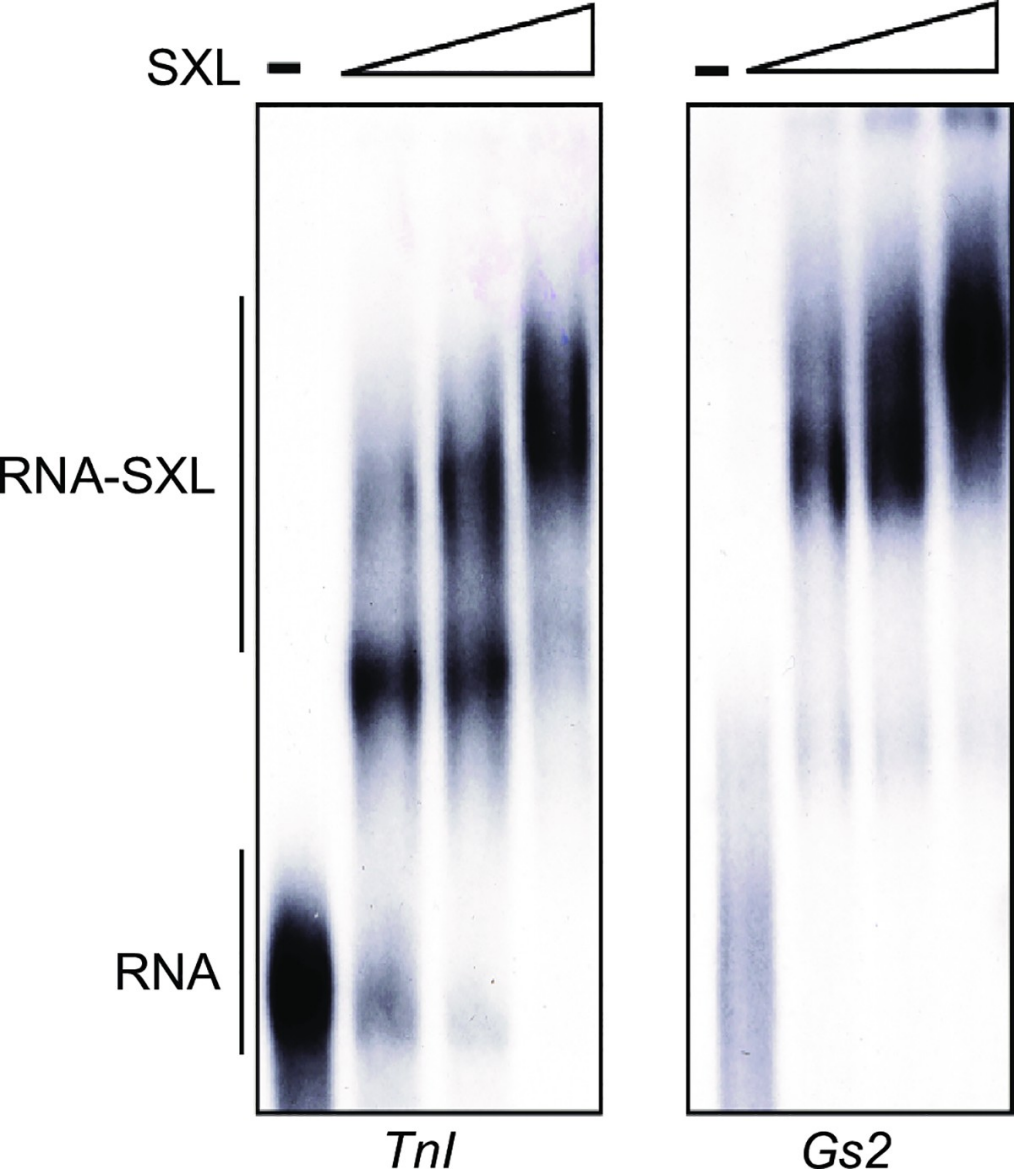
Binding of SXL to the specific uridine-rich sequences selected using cDNA SELEX. Electrophoretic gel mobility assay was used to determine binding affinity of SXL with the sequences identified in the 5’ UTR of the *Troponin I* (*TnI*) and in the 3’ UTR of the *Glutamine Synthetase* (*Gs2*) transcripts. No SXL is shown by the–lane, and increasing concentrations (three-fold successive increase) of the recombinant GST-SXL protein are shown by the triangles. The–lane shows the free (unbound) RNA, and RNA-protein complexes are shown under various SXL concentrations (three-fold successive increase) (166 ng/μl; 55 ng/μl; 18 ng/μl). For *TnI* the RNA containing UUUUUUUCUUUUUUUUCU and for *Gs2* the RNA containing UUUUUUUUUCUUUUUUUUGU binding sites were labeled using ^**32**^P and gel purified before performing the binding assay.

Since the genomic and cDNA SELEX was intended to be able to identify potential targets of SXL using the sequence tags that flank the SXL-binding site within the 150–250 long sequence in our library, we were able to identify many genes that contain these SXL binding sites. We randomly picked two of the selected sequences from the cDNA library prepared from embryonic stages 10–15 hours, sequence 5 corresponded to the *Drosophila Shaker*/*TnI* gene and sequence 14 corresponded to the *Glutamine Synthetase* (*Gs2*) gene. Both genes are located on the X chromosome. These binding sequences are present in the 5’ UTR of the *TnI* and the 3’ UTR of the *Gs2* transcripts. To experimentally show that SXL-binding sites identified in *TnI* and *Gs2* mRNAs bind SXL, we individually analyzed these sequences by the electrophoretic gel mobility shift assay. Fig 5 shows that SXL bound to the RNAs containg the SXL-binding sites from both *TnI* and *Gs2* transcripts with high affinity (*K*_*d*_ *~* 1 nM), which is similar (within 2-3-fold difference) to that for the non-sex-specific Py-tract of *tra*, which is a known biologically relevant and high affinilty SXL-binding site [29].

We conclude that SXL binds with high affinity to the newly identified SXL-selected binding sites in the 5’ and 3’ UTRs of the *TnI* and *Gs2* mRNAs, respectively.

### *TnI* and *Gs2* show sex-specific differences in mRNA expression in adult flies

**Fig 6:**
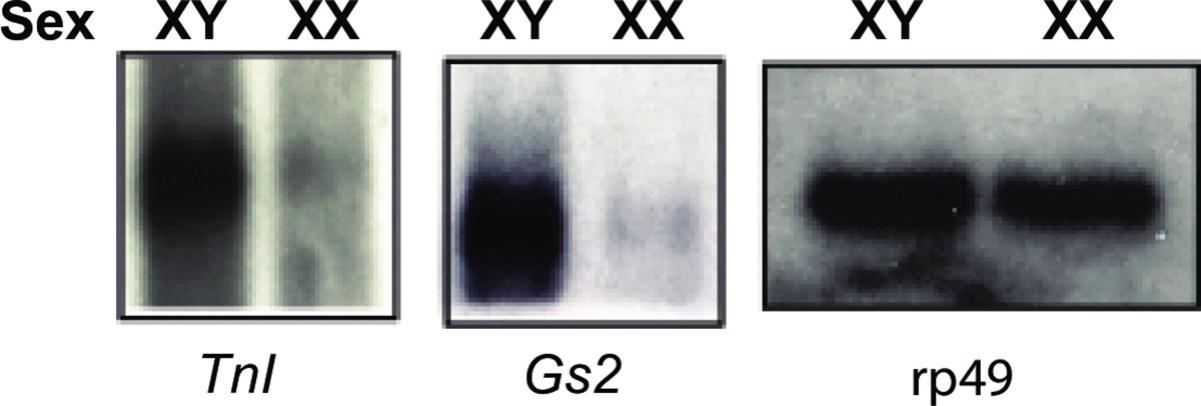
Sex-specific expression of genes containing SXL-binding sites, which are identified by the genomic SELEX. Northern analysis of the poly(A)+ RNA isolated from *w*^***1118***^ adult male (XY) or female (XX) flies. Radiolabeled probes corresponding to selected sequences (*Gs2* and *TnI*) or the ribosomal protein 49 (*rp49)* gene sequence (used as a loading control) were used to probe the RNA blots.

Since the genomic and cDNA SELEX was intended to lead us to potential targets of SXL and that SXL is present in females and absent in males, we asked if these candidates showed sex-specific differences in their expression profile or mRNA isoforms. To test this hypothesis, we performed Northern analysis for these randomly selected candidates *TnI* and *Gs2* using poly(A)^**+**^ RNA obtained from male and female adult flies. Fig 6 shows that *TnI* and *Gs2* showed sex-specific expression and these transcripts were present primarily in male flies and very little expression in females.

We conclude that *TnI* and *Gs2* mRNAs have potential SXL-binding sites and show sexual polymorphism in their expression pattern.

## Discussion

In this study, we have identified potential new targets of SXL, a subset of which may be regulated by SXL. Below we discuss advantages of GESELEX, and the biological significance of these findings. The sequences reported here would serve as a resource for those in scientific community studying sexual differentiation.

The genomic and cDNA SELEX approaches offers several important advantages. First, the genomic and cDNA SELEX libraries are much simpler than a random library (<10^**8**^ versus 10^**14**^ complexity). Thus, fewer rounds of selection-amplification could exhaust the genomic library, and would avoid artefacts from additional PCR amplifications [30]. Second, additional rounds of selection-amplification with a random library could yield super-tight binder RNA/DNA sequences. These aptamers may be useful for specific applications, such as detailed charactrization of a binding site or identification of catalytic RNAs or aptamers for diagnostics and/or therapeutics, but they may be irrelevant for biological functions which we are focused on. Thus, a genomic/cDNA library is superior for the identification biologically relevant targets for RNA-binding proteins. Third, the genomic SELEX provides a powerful alternative for the identification of natural targets for proteins for which either the binding site is unknown or the relevant genomic sequence is not complete. Fourth, identification of aptamers using genomic SELEX, which does not depend on a specific phenotype, should complement a genetic approach for the identification of downstream targets that may be missed because of redundant functions, multiple targets, lack of a detectable phenotype, or other limitations of a particular genetic screen. Finally, this reverse-genetic approach is also suitable for the structure-function analysis of DNA- or RNA-binding proteins from organisms that are not amenable for genetic manipulation or in organisms where genetic tools are less well developed.

Specifically to SXL regulation, we note that the sequences identified here from genomic and cDNA SELEX are similar to the uridine-rich sequences selected from a random pool of RNA [28] and to those present in the known targets of SXL. The latter increases our confidence that some of the sequences identified here are likely relevant for SXL regulation. Future studies should help provide a functional connection to SXL regulation. Since we have recovered some of the sequences more than once, it supports the assertion above that genomic and cDNA libraries offer simpler and less complex sequence repertoire (*Drosophila* genome is approximaely 10^**8**^) than randomized sequence libraries (typically 10^**14**^ to 10^**15**^), but provide a sequence space that is functionally and biologically more relevant to search for targets for an RNA-binding protein. We posit that our combined analysis appears to have approached saturation in terms of finding SXL-binding sites that are present in the *Drosophila* transcriptome.

In the past, the consensus-binding site for SXL was characterized by using several approaches, including SELEX from a random RNA library [28, 31, 32]. Nonetheless, identification of natural targets from the genomic database using the consensus has remained a challenging task, especially for the SXL-binding site that is a simple GU-rich sequence [28, 33]. Simple repeats are invariably masked by the repeatmasker during NCBI blast searches. It should also be emphasized that the genomic pool contained a large fraction of repetitive sequences such as the UUGUU and UUGUUU repeats, which are abundant in the *Drosophila* genome. These repeat sequences happen to be among the high-affinity SXL-binding sites. This was part of the inspiration why we undertook cDNA SELEX, which complements SELEX with genomic DNA, so that the library was not overpopulated with the GU repeats. In this sense, both genomic and cDNA libraries complement each other.

We favor that mRNAs such as *TnI* and *Gs2*, which show sexually dimorphic expression, are potentially regulated by SXL. Further detailed molecular genetic analysis in the future will be required to link these genes to SXL regulation. Both *TnI* and *Gs2* genes are located on the X chrosomosome and thus are potential targets for regulation [34, 35]. Previously, it has been argued that uridine-rich sequences, which were identified in a bioinformatics search in 20 X-linked transcripts, including *Gs2*, could mediate MSL2-independent dosage compensation [36]. However, the predominant male-specific expression of these transcripts that we observed here is difficult to reconcile with the MSL2-dependent or MSL2-independent mechanisms of dosage compensation. The MSL2-dependent mechanism equalizes mRNA synthesis in two sexes by transcriptional hyperactivation of X-chromosome genes in males, whereas the putative MSL2-independent mechanism was proposed to equalize protein expression by translation repression of the mRNAs from X-linked genes that are expected to show increased expression in females [36]. Further studies will be necessary to characterize if and how *TnI* and *Gs2* are related to the dosage compensation of X-linked genes during developmental. It is intriguing to note that the *TnI* gene belongs to a haplolethal locus of the *Shaker* complex [34], implying that the concentration of this protein is tightly regulated and may be related to critical stoichiometric requirements of cytoskeletal components. It will be interesting to determine whether the low-level expression of *TnI* in XX flies observed here is somehow related to its haplolethal phenotype.

Future studies will determine whether genes containing the SXL-binding sites, including *TnI*, *Gs2*, are biologically relevant for SXL-mediated gene regulation. SXL controls its known targets at the level of splicing, polyadenylation, or translation. It will be interesting to determine if the new potential SXL targets are regulated by these known mechanisms or differently [37], if they overlap with those identified in immunoprecipitation studies with SXL protein [42], and how they contribute to or explain the basis for sexual differentiation during *Drosophila* development.

In summary, this analysis emphasizes that the genomic and cDNA SELEX can reveal potential candidate genes that have escaped earlier attempts. Finally, such approach should be useful for the functional analysis of hundreds of the other RNA-binding proteins for which downstream targets are unknown.

## Materials and methods

### *Drosophila melanogaster* genomic SELEX library construction

The genomic DNA was isolated from *Drosophila melanogaster* adult flies using Trizol method (Sigma-Aldrich Co.). This genomic DNA (~30–50 μg) was sheared by passing through a 23-gauge needle, followed by incubating in boiling water for 15 minutes and chilling quickly in ice bath. The denatured genomic DNA was then primed for first strand synthesis using Klenow fragment of DNA polymerase 1 and 3’ random primer (SW3N7N9) followed by purification to remove excess 3’ primer. Second strand synthesis was carried out using a 5’ random primer (SW5N7N9) and Klenow fragment of DNA polymerase 1. The genomic DNA with annealed 5’ and 3’ random primers is then size fractionated on a denaturing gel and 150 nucleotide long fragments were eluted. Next, the DNA fragments from above were PCR amplified using Taq DNA polymerase and a 5’ primer (5LN7.1 220 μM), which adds a T7 promoter to 5’ end of the DNA, and a 3’ primer (3N7.1 220 μM) that binds to constant region of the 3’ random primer. PCR reaction was carried out as follows: 93°C for 30 seconds, 55°C for 30 seconds and 72°C for 45 seconds for a total of 12 cycles, followed by 7 minutes at 72°C. The PCR amplified genomic DNA with 5’-T7 promoter was purified by treating with Proteinase K, phenol:chloroform extraction and precipitation using absolute ethanol. This is the pool O genomic DNA library. The purified PCR DNA was then used for T7 transcription to generate RNA using T7 RNA polymerase by incubating the reaction mix at 37°C for overnight. After incubation, the reaction mix was treated with DNase RQ RNase free (to digest the template PCR DNA). The transcribed RNA was separated on a denaturing gel and stained with 0.1% methylene blue. The visible RNA bands were eluted from the gel using 1X Proteinase K buffer, phenol:chloroform purified and precipitated using absolute ethanol. The purified RNA (300 ng) was then used for binding to the recombinant GST-SXL protein. RNA was heat denatured and allowed to bind the GST-SXL protein (quantitated using bovine serum albumin as a standard) in a 100 μl binding reaction at 30°C for 30 minutes [38]. The bound protein-RNA complex was passed through a nitrocellulose filter and the filter bound RNA-protein complex was eluted. RNA was recovered from the filter by digesting with Proteinase K and the reaction mix was purified using phenol:chloroform and RNA precipitated using absolute ethanol.

The purified RNA was then allowed to bind 3’ primer at 42°C overnight and the annealing reaction was precipitated using Isopropanol. This 3’ primer annealed RNA fragment was then reverse transcribed into a cDNA using AMV reverse transcriptase at 42°C for 60 minutes. The cDNA was then purified using phenol:chloroform and precipitated using absolute ethanol. The cDNA was then used in SELEX PCR using 5’(5LN7.1) primer and 3’ (3N7.1) primer as described above. The amplified PCR DNA represented the pool 1 DNA. Purified PCR DNA (pool 1) was then used for T7 transcription to generate RNA using T7 RNA polymerase by incubating the reaction mix at 37°C for overnight. The transcribed RNA was purified and ~300 ng was used for second round of binding to GST-SXL protein (~160ng). For the next round of RNA-GST-SXL binding the RNA concentration was reduced to 100 ng and GST-SXL protein a ~160 ng. From round 4–7 the RNA was transcribed using radiolabeled nucleotide and ^**32**^P labeled RNA at 20K cpm was used for binding with 160 ng of GST-SXL protein, and for the final round of binding 3000 cpm RNA and 55 ng, 18 ng and 6 ng of GST-SXL protein. The pool of cDNAs from the last round of SELEX were cloned in pAMP plasmid vector (GIBCO-BRL/Life Technologies) and sequenced.

### *Drosophila melanogaster* cDNA SELEX library construction

Total RNA was isolated from the various life cycle stages of *Drosophila melanogaster* (Embryonic (0-5h, 5-10h, 10-15h, 15-20h, 20-24h); Larval (I,II,III); Pupal (I, II,III); Adult) using Trizol reagent (Sigma-Aldrich Co.) [39]. Subsequently, RNA isolated from individual stages of the fly was used to isolate and purify PolyA+ RNA using Oligo-dT affinity column. The poly(A)+ RNA was hybridized to the 3’ random primer (SW3N7N9) for first strand synthesis using reverse transcription. The cDNA was purified using phenol:chloroform extraction and resuspended in water. The cDNA was then annealed with random primer (SW5N7N9) at its 5’ end and Klenow polymerase was used to extend the fragments. The library was size selected (220 to 350 basepairs) and was PCR amplified using the 5’ primer (5LN7.1 220 μM) and the 3’ primer (3N7.1 220 μM), as dicussed above. These DNAs represented the cDNA pool libraries from each developmental stage. Subsequent steps for transcription, protein binding, reverse transcription during cDNA SELEX cycles, and sequencing were identical to those for the genomic SELEX. Protein concentrations used for binding were as follows: the first and second rounds of binding at 1:162 dilution ~9 ng (GST-SXL) with 150 ng RNA; for the third and fourth rounds of binding at 1:324 dilution ~4.6 ng (GST-SXL) with 15 ng RNA; and for the fifth and sixth rounds of binding with radioactive RNA at ~1.5 ng and GST-SXL at 1:648 dilution ~2.3 ng.

### Gel shift assay

Electrophoretic mobility shift assay was performed as described in [28, 40].

### Gene cloning

*TroponinI* and *Glutamine Synthetase 2* gene sequences were amplified from the EST clones obtained from Research Genetics (Bloomington, IN).

### Flies used

*Drosophila melanogaster* Oregon-R flies were raised in corn-meal Agar medium at 25°C incubator. etc.

### Isolation of Poly A+ RNA from *Drosophila melanogaster*

Total RNA from the flies were isolated using Trizol reagent (Sigma) according to Chomczynski, P, 1993. From this total RNA, PolyA + RNA was isolated by passing them through an oligo dT column. Northern blots containing the poly A+ RNA from various flies were generated using standard protocols [41], and blots were hybridised using radiolabeled probes as indicated in the text.

### PCR primers used for SELEX

SW5N7N9 (A-ran) BamH1–5’-AGGGAGGACGATGCGGATCCNNNNNNNNN-3’

SW3N7N9 (B-ran) Pst 1–5’-TCCCGCTCGTCGTCTGCAGNNNNNNNNN-3’

5LN7.1–5’-GAAATTAATACGACTCACTATAGGGAGGACGATGCGG-3’

3N7.1–5’-TCCCGCTCGTCGTCTG-3’

## Supporting information

S1 Table Sequences of the SXL-slected clones from the genomic SELEX library. (DOCX)

S2 Table Sequences of the SXL-slected clones from the cDNA SELEX library. (DOCX)

S3 Table List of genes or transcripts identified for the SXL-slected clones from the genomic SELEX library. Transcripts, if any, immunoprecipitated with SXL protein in primordial germ cells of *Drosophila* embryos [42] are indicated with an asterisk (*) sign. Symbol (#) indicates match in antisense strand.
(DOCX)

S4 Table List of genes or transcripts identified for the SXL-slected clones from the cDNA SELEX library. Transcripts, if any, immunoprecipitated with SXL protein in primordial germ cells of *Drosophila* embryos [42] are indicated with an asterisk (*) sign. Symbol (#) indicates match in antisense strand.
(DOCX)

S1 Raw images (PDF)
