# Supplementary material for: Genomic and cDNA selection-amplification identifies transcriptome-wide binding sites for the Drosophila protein sex-lethal
Source: PLoS One. 2021 May 24;16(5):e0250592. doi: 10.1371/journal.pone.0250592 (PMC8143406; doi:10.1371/journal.pone.0250592)
Supplement: S1 Table — (DOCX) [file pone.0250592.s001.docx]

Adult_A2

TCGAGGGCCATTAAGTTGATGTGGGCTTACAAGTTAGTACAAATTGCTACACTT TAATGCTTAATTGGGACAAAATGGATCTTTGCGTTTGCTATATGTCCGTGTTTT TTTTTTGTTTTGTTTGTTTGGGGGTAATTAACCTAGATGTGGC

Adult_A4

ATATGTTTTATGTTTTATGTTTTATGTTTTATGTTTTATGTTTTATGTTTTAT GTTTTATGTTTTATGTTTTATGTTTTATGTTTTATGTTTTATGTTTTATGTT TTATGTTTTATGTTTTATGTTTTATGTTTTATGTTTTATGTTTTATTTTA

Adult_A8

GTGTGAATGGTAAATAAAAGCGAGGTTTTGTTTTGGTTTCTCGTAGGTTTTTT TTTTTTTTTTTTTGGGCTGTGCGACCTTGAACCTGAAGAAGTCAATAAACTGCT CGTAATCGAGTGGCAAAAACTAATAATCCTGCGTTGACTATTCGTCACAATGTCG CANNGATCATTTCGCCC

Adult_A9

CGNATNANGCATTTGGGATCATCTATGAATGAANCCTTAGAAACCCCCAAGTATT TTCTGGATCATTCATATTATCNCAGTGAATNATGTNAGTTTTTAAAACTGAGTT ATATTATTGATATAGCTCATTTGUGTGTTTTTTTTTTTTGCGAATTAATTTCAGATGAACTC

Adult_A11

TCACATGGCGTATGGGCGATGCTATAACTACGTGTGTGTGTATCCATAAGATCG ATTAAAGCACCACACCGTCCATGTGTACGTGTTGCTTTGCTTCGTTTTTTTCTT TTTTTGGGCCATTCGCGTCGATGTTTCGTGGTGCAACAGGTTACACGATGANCA CAAAACATG .

Adult_A13

TTTAATCTTCTTGGTTCCAACCCTGTCATCGGTATTTGTCTCAAGTTTGAGCTC TGGTGGCTAAGTAGTTGATTTGTGTGTTTTTTGCTGCTTTTTTTAATAGTTTC

GTTGACTGTTTCTATGTTTAAATCATTANGTATATCTTAGTTTCC

Adult_A14

TTTTTTGTTTTTTTTTTTTTTTTTTGTTAATGTGTCCGTTTGAGCATGATTT TTTGTTTTGTATTTCGGAGTGTGGTTGTGCNAGGTTTTTCCTTTTCTTTAATA ATTTGAACCGCACAATCTCAATTCACCGAACGAGGAACCGAAAGGCTGAACAAGAA GAGCTGTTTCCC

Adult_A16

TTTTTTGTTTTTGCTATTTTTGTTTTTGTGTTGTTTTTTTTTTTGCGTCGAC TGCGCTGCCGACTGCGTCAATGTGAGCGCACGAGUGCAAGGGAGTGAGCGATAT GCAGGTGCCGATGATGCTCTTGGTCACTATCTCGTACTCCCTNTCACTTGCTGGCATTC

Adult_A17

GGTTTTGGGTTGAAGTTTAAGTTTGGGCCTGTTTCAGGGCTTGGATCGTTGTC AACATTCATAATTTGTCATCGAATGCGTTTGTAATTTGAAGTGATTGAGCATTT TTGCTTTTATGTGCGCTTTTTTTTTTTTTGTTTGCTTTGGTTTGATTTGGC

Adult_A18

GTTTTGTTTTGTTTTGTTTTGTTTTGTTTTGTTTTGTTTTGTTTTGTTTTGT TTTGUUUUGUUUUGUUUUGUUUUGUUUUGUUUUGUUUUGUUUUGUUUUGUUU UGUUUUGUUUUGUUUUGUUUUGUAAANGUUUUGUUUUGUUGC

Adult_B2

ATATAGGATATATGGGCGAGAATATAACAGCACTCCAGATGGTTCTGGTTTTAG CGAGAGTTGGCTGCTGTGCTGCTGCTGGCAATTAGTTTGGCTCTTCCCCTCGTT TTCGTTTTCAATTGCACTTTTTGCTACTTTTTTTTTTGCTTGATTTTTCTTCT TGCTTACTCTTTTTCTTTTTTTTTCTTGCTATGTTTTAGACCAC

Adult_B4

CGGGCTGTGAGCGGCTGTTTTTCACGGCAACGTGGAATTTATTTATGATCTTTT ATATTTGGCATGCGGCAGTTGGCTAAAAAAUATACATTCGAAATGTATTTCAAAA AATTTTCCACCCACTTTTCCTTTGTGCATATATTTGCTGATTAATGACCGCATC GCGGAAAAAGGGAAATAAATTCGTTTTTTTTTTTTTTTGGCTTTGUCAAATTTT GTTTGGTCTATTTGTATTTGTGTTGACTTTGCACTTC

Adult_B5

CNANNNNNCCNACNANNAGGANGGCCAATCNGGTTTNNGTTTTTGNCTTTGGT TNCTTTTTGGTTTTGGTTTAACCAAANNTACCAATTNCCTNNCTTGGCTANNNC TNGAANCTTTGGTTTTTTTTTTTTTTTTTTTTGTTTTTTGTTTGGTGTTCTC TTCTTTTCTTCTTCTTTTTGCTTTGTTTTTCGCTGTTCTTGTTATACAGAACG TTTAACGGTTGTTTTTTGTTTGGTGTTCTCTTCTTTTCTTCTTCTTTTTC

Adult_B6

CACTGGTCGGCGTCTGACACGACGGTGAAGTATGCATTGTTCCGCCTAGAAGAG AGTCTTGGTCTTTTTGACGGTGAGCGCGCAATTCGCGTTGGCTTATTTTAGAAC GATCGATTTGTTCGTTGTCTATTTGTTTTTGTTTTTTTTAGTTTTACTTTATT TTTCCACACACAGAAAATTTTTTGGTGTAGCAATTTAACATTGCCAATAGCGTCTCTTGAGC

Adult_B8

CATTCGAGTGGTAAACACAATATTCATGTGCGCGTTATCTGCAAATATCACCGATAAGCATGTAAATCCCATGACTGCAAGTGATTTTTTGTTTTATGTTTTTTACTCCTTTTTTTTTTTTTGTTTTAGCAGGGGAGTGGTACATATATTATTTGGAAACCAGTTACCCCAACTGCTGGACTTAAGTGGCGCAGTCGAAGCCATTTGCCAACCGAAATCAC

Adult_B9

CAAATGTGTGGCATGCGGTTAGCTCAAAATTCTGGAGTCGGGAAATGGAAAAAC GTTCTGTGGTTTCGTGGCACTTAGAATTTTCATGTGAGCGTAACCGAACCTCCG AAAAAAGGGTAGCTACATTTTAATTAACCAGTTGCTGTAATTTGTGGCCAGCCAA ATCTTTGTTTTTTTTTTTTTGTTTTTTCCGTTTTTCTGTTATTCTTTTCATACTCCAC

Adult_B11

CTGTCAGGGTTGTATGTATGTATGCACATATGGTAACCGAGATTATACAGGTAC GTGTGCTTGTGTGTGTGTGTGCGAAGGTGCGGCTGCTCGCTGTGGTTGTTGTT GCTTTTATAGGCGATTTATAACGTTGACTTCGTGTGGTGTTTTTTTTTTTGTA TTATTTTGTTGATAATTTTTGTTGTATC

Adult_B12

TTCGTGTAGATTATGTGACGTGTTGCCTAGTGTAGCCAGCGTTCACTGTACTAT TGGAACTAGTTGCTGTTAGTTACAGTTTGCTGTTTTTCTTTTTTTTTGCTGTG TATTTTGTCGTTTGCCACTTTACCTGATTTCCATCGTAAGTCCAACTACCGAAT TTCATTTCGCAATGTTGGTCATCAAATGGGAACCACGTGATGTCC

Adult_B13

ATTTATTTGGTTTTTTTGTTTTGTTTTCTTTTGCTAAAATGAATATCGAAGAA CTTTCGCCAACTGATCGGTGTATGGATTTTGCGTGTATTTCTCATCTTCGGCTT TTATTTAGCTGAATTTTAAACTTATACGCTTCGGTTAATATGGGTGATTTTTTT TCATTTTTGTTTTTTTTTTCGGGTAAATTGTAAGTGGCGGCGTAGGTGCCGTGAATGAATAC

Adult_B14

TTTTTATTTTTCTTATTTTTTTTTTTTTTTTTTTTTTGTTTGTTTATTATTT CTATGATGTTGTTGCTGGCCACGGAGCACTTTCAGCACTGATCGTTGAGCTAAG GTTTGAACACTAACACTTTCGTTTTACAAAAAATGCACTAAAATAGCTTTAGACC CTGTTCACAGTGCTAGAAATGCGTGGTGTTATATAGAAATAGTTGTGGAAATTG CGATTGTAATTGATTTAGCAATTGATTCTCTAGGCC
