# Supplementary material for: Genomic and cDNA selection-amplification identifies transcriptome-wide binding sites for the Drosophila protein sex-lethal
Source: PLoS One. 2021 May 24;16(5):e0250592. doi: 10.1371/journal.pone.0250592 (PMC8143406; doi:10.1371/journal.pone.0250592)
Supplement: S2 Table — (DOCX) [file pone.0250592.s002.docx]

0-5h­_1

ATGGGAGGACGATCGGGTCCTGTTTTTGTTCTGTTTGTTTTAGGAATTACACTAATAACTACATAGTTTCGCTTGTGTATAACTTAAAGCCTTTGTGCATCGATTTCCGCTT

0-5h­_4

TAGGGAGGACGATCGGATCAAATAGCACCTATCAGTTATTATTATTATTTATTATTTTTTGTATTACATTTATTTTATTTTGTTCTATTAACTATATCCCATATCCAAATTCACCTGAATCTAATGAATT

0-5h_9

TAGGGAGGACGATGCGGATCCGATCACTGGACGATGGGAACTAAAGAATTTCAAGAGTACCTAGACTTACCCTCCATCCCCACCATAGTAATAAGCGAGAGATGATTATCTTTATTTTTTGTATCCACTAGTTTATTA

0-5h_12

TAGGGAGGACGATGCGGGTCCTAGATCGCAGGTTCCATTTTTTGTCACTTGCAACCATAACCTTTGTGTAACTTAGTTATTTTTTTTTTTTGCACATCTAAGTCAAGAATTCTCGCAAGGTTCTCTAAA

0-5h_21

GATCGGATCCTTCTTTTTTTTTTCATATTTTTGTTTCACTTTGTTCATAATTACCTCGTAATTAAAAAATAATACATAAGGTTGCTGACGAGTTGTACAGACGCTAGTGTGTTGTTTGTGTTT

0-5h_32

GATTTTTAAAATCATTTTTGATATTTTTTTGCTTTGTTCTTTTTTGTATCTTTATATCACACGTAACGTAAGTCCTGTAGTAGATCTTATATCGTTCGTTAATCGCATGAATCTAATTGCGTTGAAAAAATAGAAATTCATTTTTCTCGACGTCAGATC

0-5h_35

GGTTATAATTGATTTTTTTTTTGTTTGTTTCCTTATACGTTTAGGCTCTTGGAAAAAAAGGAGTAAGTAGGTAGTATTNCGTTAAAGTGCTAAAA

0-5h_37

TGCTGTTGGCCTTGTGGGAGCCCTGTCGTGNNTTAGAATTCNCCTTACTCATTTCCTTTTGAGTTTTTGTTTTTTTCTCTAAAAGTTTAGATGTTTGCTGCTGCAATGTTTCGCGCGAAACTTTGTACCTTTNTAATGAATTCAGACGAC

0-5h_43

TTTAGGAGGGGAAGGTTCTCTAAAGTCCAAGTAGGTCCTATCCATTTTTGTGTTTTCTTCTCTTACTTGCAGATTTTTTTNNNTTTTTTTTTTTTTNNTTTTACAACATTCTTTTTTTTTGCTGTTGATTTTTTNGA

5-10h_1

GCGGCGAGATAATGTAGCTCCAGACAAGTGACAGGGAAGGGGTACTGGAACGAATCTCCTGTTCTTGCCTAACTATTTTACTGTTTTCCCTTGTGAATTTCGTCGAGTTATGCGAGCATTGCTTCTCT

5-10h_2

CGGATCCATTAGATGGTTATTATTATTTATTATTTTTTCTATTACATTTATTTTATTTTGTTCTATAGCTTTTATTCTTATATCCAAATTCACCTAGATCTAATGAATTAGAAAATATCAACTTAAATTCAATAAATTGAAAA

5-10h_5

CGGGTCCTAGTGTTTGTGTTTGTGTTTGTGTTTGTGTTTGTGTTTGTGTTTGTGTTTGTGTTTGTGTTTGTGTTTGTGTTTGTGTTTGTGTTGTGTCTGGCTTTGTGTTTGTGTTTGTATTTGTGTTTGTGTTT

5-10h_7

CGGATCCACTTTGCCTGCTTTTGCTTTACAGTTGTCCTGGGTTTGTAAATGGGTGTTCTTGATGTTTTTTTCTTTTTTTTTTTTTTTCCATTGGATAGGCAAAGAAGAAGAAAGAAGAAAAAATAGAAGTGAAAGAAAAGAAAACAATAGAAGGTAGAAGAATAA

5-10h_9

CGGGTCCAGATTAGATGGTTATTATTATTTATTATTTTTTCTATTACATTTATTTTATTTTGTTCTATTAACTATTATTCTTGTGTGCCAAA

5-10h_16

GGACATCGGATCCACAAATAGCACCTATTAGATATTATTATTATTTATTATTTTCTATACATTTATTTTTATTTTGTTCTGTTAACTATATCTTATATCCAAATTCAATCCTAAATCTAATGAGT

10-15h_1

AGCTATAGGGAGGACGATGCGGATCCGTTTTATTTTTTTTGTGAGATTTATTTAGGGCTTTTTTTCACAGAAGTTTCTACCCGCTATTGAATTAGGAGAG

10-15h_3

TAGGGAGGACGATGCGGATCCAAATTGCCTGCCTGCTACTGTAGCTTTTGACAAAAAGTTTTGCTTTTGACTTTTGCCTTTGTTTTTCGGGTTTTTTCTTTTTTGTTTTGACTTGTTTTA

10-15h_4

GATGCGGATCCCTTAGAGCCCGGCAGAACTCTCGGTTAAGCTCTGCCTGGGGAGCAATGAAAGTACTTTCNNNGCTTGGCCCTGTTTTT

10-15h_5

CGGATCCGTTCATTCTTTTTGTGTTGTTCAGTATCATCCGATTCGTGTGATCGACAGCATAGCCGAATTTTTTTTTTTTCTTTTTTTCTAATTCCTTAAGTAGCCAAAAACACAAATCAAAATGGCTGATGATAGGCTAAGAAGGCTAAACAGGC

10-15h_7

CGGATCCCTTTGTGTTGCCTGCAACTGCAGTTGATTTGTTTTTNTTTTCGTTTTGGTTTTTTTTTTNNTTTTTGGTTTTATATTTTCAAATTTTCTAGCA

10-15h_8

GATGCGGATCCATTATATCACCTATTAGGTGATTATTATTATTTATTATTTTTTTCTATTACATTTATTTTATTTTGTTCTATATCTATATCTATA

10-15h_9

ATAGGGAGGACGATACGGATCCCTTTTGTTTTTTGGTATTTTGTGTTTTTATTTGGCTATCAGGTGATGGGGATCGTATCAAGTGGCAGCATCGTGGATCGGATGGGCGGCTCTATCCGGACTTC

10-15h_10

ATGGGAGGATGATGCGGATCCAATTGCCCTACCAGCAACTACCCTGGCTCTGAGTTTGGACCGTCGCGTCT

10-15h_14

GGATCCAGTTTCACAGTTTTTTTTTTNCTTTTTTTTTTCGTAGCTGATGTTACTTAGGTTTTAGTATGAGTTTTTTNTTTTGTTTGTTGACCAATCAAATATATAGTAATGTGGTCAGCGTAATGACGATGAAGTGAAGAGGAGCAAGGACCC

10-15h_18

GGGTCCCTTGGTTGGTTGTTTCGTTAATGGGTAGGACACAAAGCAAGAGTTTAATTGAAATACTGANNCGTTTNGAGAATGTATATATGTATAATAGTGTTCACATAACAATTGTTTTTTTAGTTTCC

10-15h_19

GTAGTTTGTGGGAGATATGGGCAGCCTTGCGAACAAACATTTTGGTTATTCGTGCTTCGCAATTGCTGGCTTTTGGAATTGGATTAATTTTTTTTTTTTTTTTTTTTTCTTTAGAGTTCGAT

10-15h_22

CACCTATTAGATGATTATTATTATTTATTATTTTTTCTATTACATTATTTTATTTTGTTCTATTAACTATATCTATATCCAAATTCACCTAAATCTAAGGCTTAAAGAATATAACTTGAATTCAATAAAGTTAAA

15-20h_1

GGATCCTGAAATGGGGTTTGTCTATTGCACCGCTTTTTTCGGTTATCTCGGATATTTTTTTTTTGTTTTTCTTCGGGTTTTCGGGTTTTT

15-20h_3

GATCCACAATAGCACCTATAGATATTATTATTATTTATTATTTTTTCTATTGCATTTATTTTATTTTGTTCTATAACTATACTATATCCAAACACCTCAATCTAAGAATT

15-20h_6

TCGGTTCCATATTATTGATTCGTTTCACTTAAATGCACTTTGATATTTTTTGTAGAATTTTTTTTTGGTTTCTTAATGGGAAAAATACTTTTTACAGGCCCGATACCAGTTTCAAATTTCT

15-20h_9

GGATCCAGCTGGCATGTTGACTACCATGATTTACTTTTTATTTTATTTTTGGGTAGTTTTGTTCCTTTTGGGGCGTGGACCTGGTTGGATTGACGTTTTGTTTTCATATTTT

15-20h_10

CGGTTTTTTGTTTTTGCTATTTTTGTTTTTGTGTTGTTTTTTTTTNNNTCGACTGCGCTGCCGACTGCGTCAATGTGAGCGCACAGTGCAAGGGAGTGAGCATATCAGGTGCCGATATC

15-20h_13

TCGTTGGGTGGAGTTTAATTGGGCCTGTTTCANGGCTTGGATCNGTCAACATTCATAATTTNNCATCGAATNCGTTTGTAATTTTGAAGTGATTGAGCCTTTT

15-20h_15

GTTTTGGGTTGAAGTTTAAGTTTGGNNCCGTTTCAGGGTTGGATCATTGTCAACATTCACAATTCGNNGTCGAATNTTGTTTGTAATTTGAAGTGATTGAGCATTTTTNTTTTA

15-20h_16

CGGTTCACTGGATGCCACTTAGTAAATCAACTTACTTGTTAGATCAAGCCGGAACCTAGGAACAACTTTACTGTTTTTTTTTTTTTTNTTTTTGTTTTAATCCCCAAATAAAACACTTG

20-24h_1

CGGATCCGGGACTAAGACACTTGCCAGTCTTTCCGTCTCTGTCCCACTTGCACTCGCGCCTGACAGTTTCTTTTTTTTTTGCGGGGGAGGGGGAAGTTGGAGCAGAGACCGGNTGCTGC

20-24h_4

GGTTTTTTGTTTTGCTATTTTTGTTTTTGTGTTGTTTTTTTTTTTTTTNNNCGACTGCNTTNCGACTGCGACAATGTGAGCGACAGTGCAAGGGAGTGAGCGATATCAGG

20-24h_7

CGGATCCAGTGTTGTGCTGTGTTGTGTAGTGTTGAGTTGTGTTGTGTTGTGTTGTGTTGTGTTGCGTTGTGTTGTGTTGTGTTGTGTTGAATTCAGTCGACGAGCGGGAAT

20-24h_11

GGATCCGTTGTGTTGTTTTGTGTTGTGTTGGGTTGTGTTGTGTTGTGTTGTGTTGTGTTGTGTTGTGTTGTGTTGCGTTGTGTTGTGTTGTGTTGTGTTG

20-24h_13

CGGATCCAGAGTGCTTCTTATAACTTCTAATTTGTTGGGAGGGGACAATTTTTTTTATTGGATTTTTCTGGTAGATTTTGTCTTGTGTTGTGTTGGGTTGTGTTG

1 Larvae_1

GATCCAACTTTTGTTGTTTTTGTTTTCTGCCTTCGTTTTGGGATGTCGTTAGTCGACCAATGTGGAAATATTTATACTATATGTATATCTGTAAATGGATAT

1 Larvae_5

CGGATCCACCTCTCTCTCCCCTATCGTGTGTGGGGGGTTTTTGCATGGCTGCNTTAANNNNCTCTTACTAACTAGATTTAGCTTTTTTTTNNN

1 Larvae_11

CGGATCCCAGTGTGTTTCGGTTTTTTAAAATCTCTTTTTTCTTACATATTTGAAATATTTCTTTTTCTTTGGATTTTGTAAGAATAA

1 Larvae_12

TTCCGAAGTTGCTTCTTATNACTTCCAATTTGTTGGGATTTTACAATTTNNNTTGTTGGATTTTTCTGTAGATTTCGTGTTGCTAATGTGT

1 Larvae_17

GGATCGATTCTGTTTATTGAATTATTATGTGTTTTGTGTTTTNTTTCTGTTGTTTTGTTGTATTTGAGGGCAACTCTTTCAGCTAGNTCATTGGATGAACAGT

1 Larvae_18

GATCCTTAATGTTGGATATTGGAACTGTGCTGGTTCTGCCCATCGTTTTGTNGTTGGATTGTGTTTTTCTCCAGTTTTTT

2 Larvae_3

AGCGATCCTGATGTTTATTTGTTNTTTCTTATTTCCTGTATATTGGATGTACTGCTGGTTGTTNTTGCCGTTTNTGGTGGTTTTTATTTCACAATAAACTATTT

2 Larvae_8

TCCGATTAATTATGAACATATGTAATAGATNTTTTNNNNNNNNTATGGAAATAGTGAAATAC

2 Larvae_9

TGCGGGGCCTGATGTCGCTTACCAAAGCATTGGCTATCATCCCGAGGACTGCGTCGATTCCTTCACCGACTTTCAGGGGCATGACTGCTTTCATTTTGAGAGTATCTAGTG

2Larvae_10

GGATCCGAGAGAAATTTTGATCCTCGAGTATCCTGAAGGCTACNNNNNNCTCCAAT

2Larvae_11

AAACTAGCTGCGTTCTNNNTTNGTGTCTGCTTTTTGTTTCTCTGCAAACTTCAGGCTGCTTTCCAAATTTCTCTTCTGCTTTTTTTAGCACTTTTC

2Larvae_12

CTAGCTGCTTACNCAACATGAAATATTGTATCAACTATGGATCAGCCATTTATTATTGGGAATTTTATTCTTTTTTTTTTTTTATTATGTGAGAAAGTTTAGTATACAA

2Larvae_15

AACACAACCGGCTGGGTGTGAGCTCGTCGAGACTGCAGCG

2Larvae_16

ACGTGTGTATNAGTATGTGTGTATTCGTTGNNNNNTGTCTATATAACTTTNCCAGTTT

2Larvae_17

AAGAAATTTTATCATCGAGTATCTAANNCTCCAAATNCACCAATAAAAT

3Larvae_4

GGATTCTTTAGATGATTATTATTATTTATTATTTTTTCTATTACTTTATTTTATTTTGTTCTATTAACTATTATTCTTATATCCAAATTCACCTAAATCTAATGAATTGAAAAATATCAACTTAATTCAATAAATTNTGTGACAATTA

3Larvae_6

CGGATCCAACAACACCCAAACGACAACGCGCTAGCTGGAGTTGATAAATTCAATCTGAGTTCCTTTACTTTTTTTTTCGTTTTTNCCTCTTCCTTAGCTGTTTTTGTAGAATTTTTTTTTTCTATACTTATTTGTTTAGAGTTTAGCTATGGGATAGCCCGAAT

3Larvae_8

ATGCGGATCCGGCTACCTAATATCTGGATATCTGGAACTGTGCTGGTTCTNTCCATCGCTCTTTGTGCGCTTGGATTGTGTTTTTTTTGTTGTTTCTCTGGCGCTGTGTAGGGGGNTTTTACTTGTACTTGTGTACCTCGCTACCACGCTAGGCC

3Larvae_14

GGATCCTTTTTGTTTTCGGAATTTCTTTAATTTTTTGTATATTTTTAAACCAACCGAAGTTGAGTTTGTTTGTTGGTTTGTCGTTTGCGAGGCCAAAGAATCTAGCGTCTTAGCTGACTTCTTTGACTGACTGACTAAGCTCATNTGCTGCAGG

3Larvae_21

AGATAGCACCTATTAGATGATTATTATTATTTATTTTTTATTCTATTACATTTATTTTATTTTGTTCTATTAACTATTATTCTTATATACAAATTCACCTGAATCTAATGAATTAAAAAATATCAACTTAAATTCAATAAATTGAGAGGTGATAACAAATTTATTTTCTGTATTCGG

3Larvae_22

ACTTTGATGGGTAACCCCTGCCATGTGTGCTCTGCCACGTTTCATTCATCTTTGAGGCGCTCGAGGATCGATTTATTTTGAAAACTGTACTACCGGATCCCTTNNTCATCTGCTCTAATTATAAATATAGTTTTGTGTATCTTTTTTATTATTTTT

3Larvae_25

TTTTNCTACTTTTGTTTTTGTGTTGTTTTTTTTTTTNNNNGACTGCGATCCTGCCGGCGATCGATCCGACAATGTGAGCGCACGGGTGTAAGGGGGGAGCGATATGCAGGGATCCCGAGAGACCTCTGGTCTATCTCGACTCCTCTCGCGATCGGCACAGAGA

3Larvae_28

AAAGCGCCTCCGATGAAAGCGTCTCCGGTGAAAGCGCCTCCAATGAATCTTGATCATTTAGTTAACAGTTTTGTAATTTTTGTTTTTATAAATAGTATTTGTTGTTATGGCGAAAAGTCTTAACATTATACGATATTTGTGAGCGTTGAATTCAGACGACGAGCGGGAAGGTAGTAGTAGTATGTGCACCGGAT

3Larvae_32

GCACATTCCTCAACGAGACAGCGTGTGTGTGTTCCCTTTTACACAATTTTTTTTTTCTCGTGTGTGTAATTTGTAATCTGTATTCGTGTTTTTCGTTTCACCACATAATTAATGTTATTTTTGTACGGAAAAACCCGTCTGCAAAACTAGAAAATAATACACAAGGCGATCAGACGACGAGCGGATGATGATGA

3Larvae_34

GATGCGATTAAATTATTTACAAAAGAACAAACTTATCCTTTATTATCTAATTATTTAAGATGTAATATTTCTCCAATTTTTTCTTTATTTTTATCATTATTTGTTTGAATATGTATGCCTTTTTTTTTTGAAAATTATATTCTTTTAATTTGGG

3Larvae_35

GCCGGCCTTGATCTAGGTCATCTGCTCTAATCATAAATATAGTTTTGTGATCTTTTTTATTATTTTTNTTCGGCAATGGAATCTACTTAGTTTCGAATTCATAGACCATGATGTACAGTATAACGAAATTAAGAACAACATCGGAATCAATGTCGTGGAATTCAGACGACTAGCTAGGGAATGATGATGA

1Pupae_1

GGATCCGATATGCCTGTTCCCACTGTACGTCCCTGTTTATATAGGTGTTCTTTTTATTGCGTGGTACAGATTTTATTTTAATGCGTAATTCGAGATTGAGCTGGAGCAGTATTAGCGTTAGCAGCAAAGCTTACTATACGTAATAATGGAGAAGGTAATGCA

1Pupae_2

GGGTCCGATGCTCTAAAAATAGTTAGATGGCTGCACTAGTGTGTGGGTGTAGCGTGTTTATACCATGATTCTAATTTATATCCATCGACTCATCGTTTTCNNATTTTGTATTGGATATGCTTCGTGTTTTTTNTTTTCTGTTCGTTTCTCGGGGGGGAGCTGATCA

1Pupae_11

ATGCGGGTCCGTTTCGTGTGTGCCGCGATTTATTTTCCTTTTTTTTCCAATTTTTTTTTTTTTTNNTTTTTTTACGAGCTCCGGATCCTTTTTGTGTAGCAGTGGCTCAACAATCGAATTGAAACAACAACATTTTT

1Pupae_21

TTGTCGAAGCTGTGGTTTTTGTTGCCATTTTTTGCGCACAGTTGTTGCGTTTCATCCATTTATTGTTCAATTTTTGTTGGTTGTTTTTTTTTTTTTTTTTTGTATGTGCCTGTAAGTTGGAATGGACGTACTACACGGACATGAATTCAGACGACGACG

1Pupae_28

ACTACTCAAGGATTATTTTTTTTTTTACAGCTTTACTAGGAATCTATTTTACAATTCTTCAAACTTATGAATATATTGAAGCTCCATTCACTATTGCAGACTCAATTTATGGATCAACATTTTTTATAGCAGCAGGTTCACAGAATTCAGAGCAGCAGCGGGAATGATGATGATGATGTNACCCGGGAATCGACGTAC

1Pupae_32

CGATTCGGGAATGAGGTCACTGGTGTGAAGGGAATGAGTTGTTGTTGTTGTTGTTGTTGTTGATTGCTCCTTGGGATTGAGCATATTTTTTTTGTCTCTTAGAATAGAACTTACTTTAGTATTATTACTATTTGCACTAACCGAATCCAGACGACGAGCGGGAATGATGATGATGATGTCGACCCGGGAATCGACGTACTGCAGCGTACAG

1Pupae_33

TTGTGGTGCGATTAAATTATTTACAAAAGAACAAACTTATCCTTTATCATCTAATTATTAAATATATTATATTTCTCCAATTTTTTCTTTATTTTTATCATTATTTGTTTGAATGTTTATGCCTTTTTTTGTAAAATTATATTCTTTCAATTTGGGTGGTAGAGTTCAGACGACGAGCGGGAATGATGATGATGTCGA

1Pupae_34

TGTGGGTTCTTTATTTTGAAGGGGTTGATCTTGACTTGGGTGCTGCTGCTATCGCTTCTAGATGTTGTTACTGTTGGCTGGCTTTGTGAATGTTGTGGCAAATTGATTTTTCTTTTTTTTTCATTTTTTTCAANTTCTCATCTTTTCTTCTGGTTTTTCTCCGTGTGGTTGAATTAGAGAGAGCGGGAATGATGATGATGTCG

2Pupae_3

CCCGGCCTGGTCCCGGAGCTTAACTTATCACCCATTCCGCATTGTGAATCTGAATGTGTTTTTTTTTTTTTTTTTTTTTTTTTTTTTATCGCGGTTGAATTTACTTTGTGATTAGCTAAATTTGTAACTGAACGATCGACTGAT

2Pupae_6

CGGGTCCTAGCACGATTGCGCAATTGTAAATCGTAATCGTGTTGTCTTCGCAACCCCAAAGAGTGACCNGGACAT

2Pupae_9

AGTTCCTCGGATATGAGGTTATTTTCGCCAATGATGATTATATTCCTCCATGTCCGCCATGCTGAAGGTCCGTGAAATTTTGTCTGTTTTGAATTTGTCTTCCTCGCAAACGAAAANGTTGTTTTTTTTTTTTATCCGTGCAGAATTCACGACGACGAGGGATGATGATGATC

2Pupae_12

CATGTTAATATGTTTTCATTTTTTTTTTATATATTCTCTACGGGTTTAACTGTTAGTTATTCATTTCGATTAGTTTATTATTCAATACCGGTGATTTAAATTGCGGTAGATTGAATATTTAAATGATGAAAGTTGGATTTTTACTTCGTGGTATAATAGGATTATTAACATAGATATGAGACTGATCAGAG

2Pupae_13

CATGCTTCTTTTGATTTGTAATGTTTTTCTTTACATTTTTTGTATTATTTTTGGTTTTATTTTTTTAGTTGGTTCTGCCCCTGCAGACCGGCTGCAAATGGCCAAAAGGTTCGTCGACAGTTTTGTGTTTTTTTCAGCTGTTCGACTGACNAAGGCAGAATTCAGA

2Pupae_15

CGTTTTTATTATCGTGTTGTTAGCTTGGTCCCAGAGCTTAAACTTATCACCCATTCCCATTGTGAATGCTGAATGTGTTTTTTTTTTTTTTTTTATCGCGGTTGAATTTACGTTGTATTTAAACTTAATTTGTAAACTGAAACGAAA

2Pupae_17

AGATCGGATCCTGGATGGCGATTTAAGGTTGCAAAACTAACTGCCGTTCTTGGTTNGTGTCTGCTTTTTNTTTCTTTGCAAACTTCAGGCTCGCTTTGGAAAGGGCCGC

2Pupae_18

CGGATCCCGGGCATTTTGTTGGTGTTGTATTTTATGGATTTCTCTCGAGAAAAAAGTTT

2Pupae_19

CAGGATGCGTAGGAAAACACGAATGTGCAAAGGGGTTTTCTCTTTTTTTTTTGTTTTATAATTTCTGTTTATCTTAAGTTGTAGGTTGTTATTTGTTGTTGTTGATGGCGCGCAGGGAATTCAGACGACGAGCGGGAATGATGATGATGATGTCGACCCGGGAATTCCGACCGTACCTGCAGGCGTACCAGCTTTCCCTGTG

2Pupae_22

AGAGATAGATGATTCCGATCTGGATCTCGATCTGGTAATCATCACTCTCTGGGGGTTCTTCCCTCTAGTTCTTCAACGGACACGGATATAAAAATAGACACGGCTTGTTTGTTGTTTTTAAGTTTTTTTTTTTTTGTTGATTTTATAGGTGTTGAATCACGACGACGAGGGATGATGATGATTCGGACCGATCGACGACTC

2Pupae_24

ACCTAAATGGGATAGTTTTGTTTTCCGGCCTTTTAGTTTTTCGGTGTGAGTTTGTGAGCTTGTAACCGTTCGAAATTTTGTGCTTTAAGTTTTTGTTGGACAAGTCGCTGCCATGTTTCACCCTGAATTCGAACGAGGTACGACGACGAGCGGGAAGTAGTAGTAGTTGNCCCGGGATCGA

2Pupae_25

CCTAAATGGGATAGTTTTGTTTTCCGTCTTTTTTGTTCTTCGGTGTGAGTTTGTGAGCTTGTAACCGTTCCAAATTTCGTGTTTTAAGTTTTTGTTGGACAAGTCGCTGCCCTGTTTCAGATCAAGAGGTAAGTATTAATAGAAGAGCTTTGGGTGGACTTTGAATT

2Pupae_28

GCACCTATTAGATGATTATTATTATTTATTATTTTTTCTATTACATTTATTCTATTTTTTTCTATTAACTATTATTCTTTTATACCAAATTCACCTAAATCTAATGAATTAAAAAATATCAACTTAAATTCAATAAATTGGAAATGATAACAACTTTATTTTCTGTATGCACCCTATGATACGAGCAGCAGCGGAGTAGTAGTAGTAGTGACCGGAT

2Pupae_29

GTTTTTGTTTTTTTTTGTTTTTTGTTTTTGCTTTAACCTGACGTTTCTGCGCTACTACATTTTTCTCATTTATTTTTTGATGCCCACTTTATAATACGGCCGATCTCTTCTCTCCAATGACATCTTCCCAACGAATTCATTGGAGAGAGAGGTATGTAGATGGGCTCAGATCAGAGCAGCGACGGAGTAGTAGTAGTAGTTGCACCGGAT

2Pupae_33

TTCGAGATTCGTCGGCTTATTTATTTATCTCCCTATTCTCCTTATTTTTTTTTTTTTTTTGCGATTTTGGCAGTGCAAGTAACGGTGAACTAGGAAGAAGTTGAGCGTGCCGATTTCCTTTTGTTGTCTGCTCACTCACACGACAGTAATTCAGACGACGAGCGGGAATGATGATGATGTCGTCCGGATCGACGTACTAC

2Pupae_34

TGTCTGGATATCTGGAACTGTGCTGGTTCTGTCCATCGCTCTTTGTGCGCTTCGATCGTGTTTTTTTGTTGTTTTTCTGGCGCTGTGTATTTTTGTTTTCGTTGTCATTGTGCCACTCTGCCACTCTGCCACTGGAATTCAGACGACGAGCGGGAATGATGATGATGATGTCGACCCGGGAATCGACGTACTGCAGGCGTACAGC

2Pupae_36

CACTTACTTGTTAGATCAAGCGCGAACCTGGAAACAACTTTACTGATTTTTTGTTTTTGTTTTAATCCCCAAATAAAACACTTGTTTTTTTGTTGTTGTTGTTTGATTTCGCGAGTTTTTCACCCAGAAGAAGGTAACAATCTATGGGCTGTTGGTAGAGCAATTAGCAGCAGCAGCGGAA

3Pupae_1

CCTGCCTGCTGCNTATATATTTTTTTTTTCCTGCTGCATTCAACAAAACTCCGGTGAGAACGGNGGACGGAAATCTTGCACCTCAATGTTTATCACTTTTTACCCCTCNNNNTTNCTCCCTNNAAACAGCCAAA

3Pupae_2

GGACGATCGGGGAGGACGATNCGGATCCTTTGTTGACTAATTATTTAAGATATCATATTTCTCCAATTTTTTCTTTATTTTTATCATTGTTTGTTTGAATATGTATGCCTTCTTTTTGTAAAGTTATATCTTTTAATTTGGG

3Pupae_7

CCCGGGACTCGTATCGGGAGGCTCTACTTTGCNCGCGNTCCGGCTCATCGGGTGCATGGCGTTGAAAACGAGTGTTCCTCGGTTGGTTATCGTCTGTTCCCGGANCAATGCTAGTTCGTCTACTCATTCATTTTTTTCTTTTTCACTCCAATTCAATTGCTGCCAAAGTGGACTGCGAATT

3Pupae_9

CCGTTGTTATTATCGTGTTGTTAGCCTGGTCCCAGAGCTTAAACTTATCCACCCATTCCGCATTGTGATGCTGTATGTGTTTTTTTTTTTAATCNNGTTGAATTTACNTTGTATTTAAACTTAATTTGTAAACTGAAAGCAAATGCATGTGTGAATTAGCAGCAG

3Pupae_12

GGGAGGACNATNCGGGTCCAAGTTCTTTTTTTNTTTTGTTTTAATTTAATTTNCNCCACTCGAAAAATTGTGTGCGNTTTGAATACNTTTGAAATTTTCATTT

3Pupae_14

ACGATACGGATCCGAGTTGGAACATACCAAGGATTAGATAGTTATGCAGTAAGTATTGGTTTACGATGAGGAATAATTTTATTTATTTTATCAGAAGTTTTATTTTTT

3Pupae_17

GATGATNCGGGTCCGAAGTGCTTCTTATAACTTCTAATCTGTTGGGATTTTACAATTTNNNTTNTTGGATTTTTCT

Adult_1

CGGATCCTTTGTTAGTTTTTATTTATTAATTTTTATTATTTTTTTTTTAAAAAATTACTAGAAATAACTATAAAATTTAAAGTTTTAGTATTGTTTAAGATAAAAATAATTTTAATAATAGTTTATTAGTATTGT

Adult_2

CGGATCCAGTATACAGTTTCATTTTTTTTTTTTTTTTTCTCATTTCCAGTTATTTCATTCGCCTTTTCTAAGTACTAATACTGCCGTTCTCTACTCCAATTTCTGTGGAAGGATGGATTA

Adult_3

CGGGTCCGCTGCTGTCCTTATCGTTTTATTTATACAGAGTGGTTTGTATTGTTAATAATATATTACAGACTATTTTATGCAGAAATTAGACAAAAAGAATTCAAATCAGTTCTGTGGGGTTGTCGGTAA

Adult_4

CGGGTTTTTGTTTTGTTTTGTCTTTGTTTTGTTTTTGTTTTGCTTTGTTTTTTTGTTTTTNNNNTTTTTGTTTTTTGTTTTGTTGTTTGTTTTGCAGAGCA

Adult_5

GGGTCACAAATAGCACCTATTAGATGATTATTATTATTTATTATTTTCTATTACATTTATTTTATTTTGTTCTATTAACTATTATTCTTATATACCAAATTCACCTAAATCTAATGAATT

Adult_7

CGATCGGGAATGATGATNATGCAGACGACGAGCGGGAATGATGATGATGTCGACCCGGGAATTCCGGNNGGTACCTNGGCNTACCAGCTTTCCCTATAGTGAGC

Adult_8

AGTGCTGTGTTGCGTTGTGTCGTGTCGTGTTGTGTTGTGTTGTGTTGTGTTGCGAATTCAGACGACGAGCGGGAATGATGAT

Adult_9

CGATGCGGGAATGATGATGATGCAGACGACGAGCGGGAATGATGATGATGATGTCGACCCGGGAATTCCGGACCGGTACCTGCAGGCNTACCAGCTTTCCCTATAGTGAGTCGTATTAGAGCTT

Adult_15

GGATCCGGCTTTAGTGATTACGTTGTTAAGATAACAAATCTTTTACAGCTGAATTCATTTNTTCTCGTTTCATTTTTTT

Adult_16

AGATNGGNNNGGNATNTTACANAATGGAATATTGTATAACTATTGGATCAACTATTTATTATTAGGAATTTTTATTATTTTTTTATTATTTGAGAAAGTTTAGTATACAA

Adult_19

ATGTTCGATAGTTTTTTGATTATTTTTGTTTAGCTGTGTGAATTTGTATTCGAAGAGGGGTTCAGACACTAATTCGGAATTCGCTTTGAATATCATTCGTGTATTTCACATCTAAATAC

Adult_20

ATGCAGACGACGAGCGGGAATGATGATGATGATGTCGACCCGGGAATTCCGGACCGGTACCTGCAGGCGTACCAGCTTTCCCTATAGTGAGTCGTATTAGAGCTTGGCGTAATCATGGTCATA

Adult_21

ATTATTATTATTTATTATTTTCTCTATTACGTTTATTTTATTTTGTTCTATTAACTATTATTCTTATATCCAAATACACCTAAATCTAGTGAGTTAAAAAATATCAACTTAGATTCAATAAATTGAAAATGATAACAAATATA

Adult_23

ACCCGTTAGATGATTATTATTATTTATTATTTTTTCTATTACATTTATTTTATTTTGTTCTATTAAGTATTATTGTTATATACCAAATTCACCTAAATCTAATGAATTAAAAAAAATATCAACTTAAATTCAATAAATCGGAAATGATAAGAAATTTA

Adult_24

GTGCGCTGCTATAGAGACCATATATAGCAATGGTCTTTCATCAGGTTTTTAGTTTAGCTCTACTATTATTTTAGTATTTTTGTTTCTGTTTTTGTTTNGGTTTGTGGGATTTTTTTGGGAGCTCTGGGTGCCTTGAATTGATACGATGAGCGGTT

Adult_25

TAATTAATGACGAAAACTAACTAAACTTAGTTTATTTCGGAAAAATGTTTTTTTTTTTTTTTTGGTATTTCAAGCTATCTGTTTGTGGATTCGTATGACACGCGTAATTGCGTAAGTGTTTGACTGGCCGGATTTCT

Adult_31

TAGAGAGCAAATCGTTTTTGAAAAAGTCCCCAGAAAAATTTTTCGTTTTTTTTTTTTTTCTCTCCGATATAGTTAAAACTTTCTCTTATCTTTTTTCGTTTCGGATTTCTCGACCAAATTTTGAGTGTGGAGACAGACGGACGCTAGAATTCAGA

Adult_32

ATTATTATTATTTATTATTTTTTCTATTACATTTATTTTTTTTGTTCTATTAACTATTATTCTTATATACCAAATTCACCTAAATCTAATGAACTAAAAAATATCAACTTAAATTCAATAAATTGAAAATGATAACGAATTTATTTTCTGTATTCGACCCAAGGAATTCAGA

Adult_33

GTGTTTGTGTTGTTCTGTGTTGTGTTGTGTTGTGTTGTGTTGTCTTGTGTTGTGTTGTGTTGTGTTGTGTTGTGTTGCGTTGAATTCAGACGGCGAGCGGGAGGCCATAAGGATTCAGACGACGAGCGGGAATGATGATGATGATGT

Adult_35

GGACACCTTGACTTATTCCCAGCACCAGACCTAAATGGGATAGTTTTGTTTTTCCNCTTTTTTNTTTTTCGGTGTGAGTTTGTGNNCTTGTAACCGTCGAAATTTTNNNTCTAAAGCTTTTTTGCTAGGACAAG

Adult_36

GATTTTAGGTTTGCTGTGGGTTCGCGCGAATGAATCTATACAAGCTTCAAATGGAATCCCTTACAGTTTNTNCGCTTGTTTCTTTGTGTGCTTGTTTCTTTTTTCGTTTCGTTTCGTTTCTTTCGTCAGTTTGTAAGTACCATGACCGANCCA

Adult_39

ATCCTTTTTGTGATGCGATTAAATTATATACAAAGAACAACTTATCCTTTATTATCTAATTATTTAAGATATTATATTTCTCCAATTTTTTCTTTATTTTTATCACTATTTGTTTGAATATGTATGCC

Adult_40

ATCCTGTGTGTTCGTGTAGCGTGTCTATATAAACTTTGCCAGTTTGCAAAAATTTCAAATAGTTTTCTTTAACAGTTTTTGTTTTATTTGTATTTGTGTTTTTATTTGTTGTTTGTTTTNCTTTTA

Adult_41

GGTCCTTGGGTTGTGTTGTGCTATGTTGTGTTGTGTTGTGTTGTGTTGTGTTGTGTTGTGTGAATTCAGACGACGAGCGGGAATGATGATGATGTCGACCCGGGAA

Adult_42

GGTCCGTGTTGTGTTGTGTTGTGTTGTGTTGTGTTGTGTTGTGTTGTGTTGTGTTGTGTTGTTGTTGTGTTGTGTCGTGTTGTGTTGTGTTGTATGAATTCAGACGACGAGCGGGATGATGATGATGATGTC

Adult_50

GGAATTCAGACGACGAGCGGGGAATATGATGATGATGATGATGATGATGTCGACCCGGGAATTCCGACCGGTACCTGCAGGCGTACCAGCTTTCCCTATAGTGAGTCGTATTAGAGCTTGGCNAATCAT

Adult_51

GGAGTAGAATTTGCCAGCAGCTCAAATCTGTTATTTTTTTTTTTTTGTGGCGTTTTGTTAGTGATCATGTTAGCATTGTCGCGTGTAATTTTTGATTT

Adult_52

GAGACGACGAGCGGGAATGATGATGATGATGTCGACCCGGGAATTCCGACCGGTACCTGCAGGCGTACCAGCTTTCCCTATAGTGAGTCGTATTAGAGCTTGGCGTAATCATGGTCATAGCTG

Adult_53

GTGTTTGTGTTTGTGTTTGTGTTTGTGTTTGCGTTTGTGTTTGTGTTTGCGTTTGTGTTTGTGTTTGTGTTTGTGTTTGTGATAGAGGGATTCTGTCGAC

Adult_54

GGGTCCCTTCTCTTTCAGTTTTTTGTTTTTGTGAATTTTCGGTTTTTGTTGTTTTCTTTTTTTGTTGGTTTTCGGTTTTTGTGAATAACGGGCGACTTGAAAACAGAAATT

Adult_56

AATGATGATGATGCAGACGACGAGCGGGAATGATGATGATGTCGACCCGGGAATTCCGGACCGGTACCTGCAGGCGTACCAGCTTTCCCTATAGTGAGTCGTATTAGAGCTTGGCNAATCATGGTCATAGCT

Adult_58

AATGATGATGATGCAGACGACGAGCGGGAATGATGATGATGTCGACCCGGGAATTCCGGACCGGTACCTGCAGGCGTACCAGCTTTCCCTATAGTGAGTCGTATTAGAGCTTGGCGTAATCATGGTCATAGCTGTTTCTGTGTG

Adult_59

TCCTTCCCGATTTTATTGGTTTTTTTTTTTTTTGTTGACTTGCCCGCGGTTTTTGGGGACGCACAGGNAAATCAGCAGCTGAACTTAAAGCAATTAGACTAACTCA

Adult_60

GAATGATGATATCGAGACGACGAGCGGGAATGATGATGATGTCGACCCGGGAATTCCGGACCGGTACCTGCAGGCGTACCAGCTTTCCCTATAGTGAGTGTATTAGAGCTTGGGCTAA

Adult_61

TCCATAAATGGCACCTATAGATGATTATTATTATTTATTATTTTTTCTATTACATTTATTTTATTTTGTTCTGTTAACTATCATTTCTTATATACCAAATTCACCTGAATCTAATGAATT

Adult_62

TCTATTTNCNCGCTCGGTTGGGTTTTTTGTATTTGTATTTTTGTTTTTTNNTTTTTTGGTTTTGGTTTCTTGGCATGTGTTGGTTTTGTTTTTGGGAGGGATTGCTTGG

Adult_63

ATCCTGTGTGGTGTATGTGTGTATGAGAGTGTGTTCGTGTAGCGTCTCTATATAAACTTTNCCGGTTTGCAAAGATTTCAAAGTAGTTTTCTTTTAACAGTTTTTGTTTTA

Adult_80

TGGTTTTCTTTTTCTTTCTTTCTTTCTATTCTTCTGTTCTCTGCACAGTTTGCTTTCCATTTTTTCGTTTTTATTTTAGCTCTTTTGCAATTTTTTGTTTTTTCTTTGCCTTTTTAAAAGTGTATGGTTGTCGAATTCAGACGACGAGCGGGAATGCTGCTGCTGCTGCTGCTGCTGCTGCGACCGGATCGACGACT

Adult_84

CCTTTACTTTTATTGACATCATTTTGCCTTACCTCGATTTCAGAGTGCGGTGTTTAAAGCTCAACTAAAAACTAATAAAGAGTGTGTGACACAATAACTTAGGCAAGTGATTGGATTTTGTTAGTTTTTTTTTTTCATTTATGGTGGCGGAATTCAGAGCAGCAGCGGGAATGTAGTAGTAGTAGCG
