# Supplementary material for: Genomic and cDNA selection-amplification identifies transcriptome-wide binding sites for the Drosophila protein sex-lethal
Source: PLoS One. 2021 May 24;16(5):e0250592. doi: 10.1371/journal.pone.0250592 (PMC8143406; doi:10.1371/journal.pone.0250592)
Supplement: S3 Table — Transcripts, if any, immunoprecipitated with SXL protein in primordial germ cells of Drosophila embryos [42] are indicated with an asterisk (*) sign. Symbol (#) indicates match in antisense strand. (DOCX) [file pone.0250592.s003.docx]

|  | Clone ID | Transcript ID# | Gene name | Location |
| --- | --- | --- | --- | --- |
| 1 | Adult_A2 | FBtr0330387 | *CDK ap1#* | X |
| 2 | Adult_A4 | No match | *_* | _ |
| 3 | Adult_A8 | FBtr0308682 | *CG13004* | X |
| 4 | Adult_A9 | FBtr0345809 | *CG34452* | 3L |
| 5 | Adult_A11 | FBtr0112805 | *Fas3* | 2L |
| 6 | Adult_A13 | FBtr0346492 | *CR45712* | 2L |
| 7 | Adult_A14 | FBtr0334867 | *stg* | 3R |
| 8 | Adult_A16 | FBtr0309982 | *CR43626* | 3L |
| 9 | Adult_A17 | FBtr0346418 | *Arpc2* | 2L |
| 10 | Adult_A18 | No match | *_* | _ |
| 11 | Adult_B2 | FBtr0474229 | *CG46385#* | 2R |
| 12 | Adult_B4 | FBtr0333262 | *dlg-1* | X |
| 13 | Adult_B5 | No match | *_* | _ |
| 14 | Adult_B6 | FBtr0336693 | *Drp* | 2R |
| 15 | Adult_B8 | FBtr0336788 | *htt* | 3R |
| 16 | Adult_B9 | FBtr0289965 | *Or24A* | 2L |
| 17 | Adult_B11 | FBtr0343036 | *CR44723#* | 3L |
| 18 | Adult_B12 | FBtr0331284 | *nAchRα6*#* | 2L |
| 19 | Adult_B13 | FBtr0333320 | *fne#* | X |
| 20 | Adult_B14 | FBtr0346347 | *rin#* | 3R |
