# Supplementary material for: Genomic and cDNA selection-amplification identifies transcriptome-wide binding sites for the Drosophila protein sex-lethal
Source: PLoS One. 2021 May 24;16(5):e0250592. doi: 10.1371/journal.pone.0250592 (PMC8143406; doi:10.1371/journal.pone.0250592)
Supplement: S4 Table — Transcripts, if any, immunoprecipitated with SXL protein in primordial germ cells of Drosophila embryos [42] are indicated with an asterisk (*) sign. Symbol (#) indicates match in antisense strand. (DOCX) [file pone.0250592.s004.docx]

|  | Clone ID | Transcript ID | Gene | Location |
| --- | --- | --- | --- | --- |
| 1 | 0-5h­_1 | FBtr0342895 | *Sdc#* | 2R |
| 2 | 0-5h­_4 | Fbtr0100866 | *ATPase8* | Mitochondria |
| 3 | 0-5h_9 | FBtr0329832 | *hippo* | 2L |
| 4 | 0-5h_12 | FBtr0075323 | *Nrt* | 3L |
| 5 | 0-5h_21 | FBtr0345894 | *Act5C* | X |
| 6 | 0-5h_32 | FBtr0082693 | *Paip2#* | 3R |
| 7 | 0-5h_35 | FBtr0340164 | *Ran* | X |
| 8 | 0-5h_37 | FBtr0079937 | *Pen#* | 2L |
| 9 | 0-5h_43 | FBtr0301524 | *D1-RAD#* | 3R |
| 10 | 5-10h_1 | FBtr0346479 | *CR45700#* | 2L |
| 11 | 5-10h_2 | FBtr0100866 | *ATPase8* | Mitochondria |
| 12 | 5-10h_5 | No match | _ | _ |
| 13 | 5-10h_7 | FBtr0331176 | *fax#* | 3L |
| 14 | 5-10h_9 | No match | _ | _ |
| 15 | 5-10h_16 | FBtr010086 | *ATPase8* | Mitochondria |
| 16 | 10-15h_1 | FBtr0100868 | *CoIII* | Mitochondria |
| 17 | 10-15h_3 | FBtr0074280 | *eIF4H1#* | X |
| 18 | 10-15h_4 | FBtr0344877 | *dpr1#* | 2R |
| 19 | 10-15h_5 | FBtr0308237 | *Tn1* | X |
| 20 | 10-15h_7 | FBtr0344910 | *chrb#* | 3L |
| 21 | 10-15h_8 | No match | *_* | _ |
| 22 | 10-15h_9 | FBtr0087912 | *CG13159#* | 2R |
| 23 | 10-15h_10 | FBtr0310416 | *miple1#* | 3L |
| 24 | 10-15h_14 | FBtr0343562 | *Gs2* | X |
| 25 | 10-15h_18 | FBtr0346376 | *Atg8a#* | X |
| 26 | 10-15h_19 | FBtr0334873 | *Cnx99A#* | 3R |
| 27 | 10-15h_22 | FBtr0100866 | *ATPase8* | Mitochondria |
| 28 | 15-20h_1 | FBtr0082534 | *Lk6* | 3R |
| 29 | 15-20h_3 | FBtr0345419 | *hbs* | 2R |
| 30 | 15-20h_6 | FBtr0344237 | *MTF-1#* | 3L |
| 31 | 15-20h_9 | FBtr0333541 | *vkg* | 2L |
| 32 | 15-20h_10 | FBtr0309982 | *CR43626* | 3L |
| 33 | 15-20h_13 | FBtr0081246 | *Top3α* | 2L |
| 34 | 15-20h_15 | FBtr0339545 | *qvr* | 2R |
| 35 | 15-20h_16 | FBtr0347285 | *CG33939* | X |
| 36 | 20-24h_1 | FBtr0304628 | *Cpsf6#* | 3L |
| 37 | 20-24h_4 | FBtr0079026 | *Pgant5* | 2L |
| 38 | 20-24h_7 | FBtr0331752 | *sgg** | X |
| 39 | 20-24h_11 | No match | *_* | _ |
| 40 | 20-24h_13 | FBtr0086151 | *Vha16-1#* | 2R |
| 41 | 1 Larvae_1 | FBtr0305605 | *rudhira* | X |
| 42 | 1 Larvae_5 | FBtr0330019 | *Gγ30A* | 2L |
| 43 | 1 Larvae_11 | FBtr0303036 | *Bulli* | 3L |
| 44 | 1 Larvae_12 | Fbtr0086151 | *Vha16-1#* | 2R |
| 45 | 1 Larvae_17 | FBtr0346349 | *RpS3#* | 3R |
| 46 | 1 Larvae_18 | FBtr0074631 | *CG6891* | X |
| 47 | 2 Larvae_3 | FBtr0084994 | *Ald1#* | 3R |
| 48 | 2 Larvae_8 | FBtr0332935 | *CG15765* | X |
| 49 | 2 Larvae_9 | FBtr0070538 | *Ng4* | X |
| 50 | 2Larvae_10 | FBtr0075503 | *CG13445* | 3L |
| 51 | 2Larvae_11 | FBtr0080306 | *CG6770#* | 2L |
| 52 | 2Larvae_12 | FBtr0100861 | *CoI* | Mitochondria |
| 53 | 2Larvae_15 | FBtr0073435 | *Hsp60A* | X |
| 54 | 2Larvae_16 | FBtr0330225 | *BicD* | 2L |
| 55 | 2Larvae_17 | FBtr0075503 | *CG13445* | 3L |
| 56 | 3Larvae_4 | FBtr0100866 | *ATPas8* | Mitochondria |
| 56 | 3Larvae_6 | FBtr0303335 | *prtp* | X |
| 57 | 3Larvae_8 | FBtr0074631 | *CG6891* | X |
| 58 | 3Larvae_14 | FBtr0346384 | *sesB#* | X |
| 59 | 3Larvae_21 | FBtr0100866 | *ATPas8* | Mitochondria |
| 60 | 3Larvae_22 | FBtr0344542 | *SclB* | 2L |
| 61 | 3Larvae_25 | FBtr0479788 | *Ca-α1D*#* | 2L |
| 62 | 3Larvae_28 | FBtr0075503 | *CG13445* | 3L |
| 63 | 3Larvae_32 | FBtr0331719 | *CG11151* | X |
| 64 | 3Larvae_34 | FBtr0433499 | *ND1* | Mitochondria |
| 65 | 3Larvae_35 | FBtr0344542 | *SclB* | 2L |
| 66 | 1Pupae_1 | FBtr0339433 | *CG13366* | X |
| 67 | 1Pupae_2 | FBtr0333137 | *CG12605* | 3L |
| 68 | 1Pupae_11 | FBtr0073916 | *CG9411* | X |
| 69 | 1Pupae_21 | FBtr0082940 | *soti* | 3R |
| 70 | 1Pupae_28 | FBtr0100868 | *CoIII* | Mitochondria |
| 71 | 1Pupae_32 | FBtr0344335 | *eIF4G2#* | 3R |
| 72 | 1Pupae_33 | FBtr0433499 | *ND1* | Mitochondria |
| 73 | 1Pupae_34 | FBtr0303464 | *CNBP#* | 2R |
| 74 | 2Pupae_3 | FBtr0299776 | *CG42323* | X |
| 75 | 2Pupae_6 | FBtr0333345 | *Hr4* | X |
| 76 | 2Pupae_9 | FBtr0091692 | *CG33704#* | 2R |
| 77 | 2Pupae_12 | FBtr0433501 | *ND5* | Mitochondria |
| 78 | 2Pupae_13 | FBtr0085679 | *CG9717* | 3R |
| 79 | 2Pupae_15 | FBtr0299776 | *CG42323* | X |
| 80 | 2Pupae_17 | FBtr0080306 | *CG6770#* | 2L |
| 81 | 2Pupae_18 | FBtr0086701 | *Pepck1#* | 2R |
| 82 | 2Pupae_19 | FBtr0071883 | *blw#* | 2R |
| 83 | 2Pupae_22 | FBtr0089747 | *Mlc2#* | 3R |
| 84 | 2Pupae_24 | FBtr0308944 | *CG9284#* | 2R |
| 85 | 2Pupae_25 | FBtr0308944 | *CG9284#* | 2R |
| 86 | 2Pupae_28 | FBtr0100866 | *ATPase8* | Mitochondria |
| 87 | 2Pupae_29 | FBtr0333355 | *Nrv3#* | 2L |
| 88 | 2Pupae_33 | FBtr0339099 | *Snp#* | 2R |
| 89 | 2Pupae_34 | FBtr0074631 | *CG6891* | X |
| 90 | 2Pupae_36 | FBtr0347285 | *CG33939* | X |
| 91 | 3Pupae_1 | FBtr0331710 | *g*#* | X |
| 92 | 3Pupae_2 | FBtr0076150 | *CG33271* | 3L |
| 93 | 3Pupae_7 | FBtr0085352 | *WASP#* | 3R |
| 94 | 3Pupae_9 | FBtr0299776 | *CG42323* | X |
| 95 | 3Pupae_12 | FBtr0474169 | *Sdc#* | 2R |
| 96 | 3Pupae_14 | FBtr0100868 | *Colll* | Mitochondria |
| 97 | 3Pupae_17 | FBtr0086151 | *Vha16-1#* | 2R |
| 98 | Adult_1 | FBtr0100888 | *CG34094* | Mitochondria |
| 99 | Adult_2 | FBtr0343318 | *CG7536#* | X |
| 100 | Adult_3 | FBtr0300484 | *lolal#* | 2R |
| 101 | Adult_4 | No match | _ | _ |
| 102 | Adult_5 | FBtr0100866 | *ATPase8* | Mitochondria |
| 103 | Adult_7 | FBtr0333925 | *CG31324* | 3R |
| 104 | Adult_8 | FBtr0331752 | *sgg** | X |
| 105 | Adult_9 | No match | *_* | _ |
| 106 | Adult_15 | FBtr0086539 | *βTub56D#* | 2R |
| 107 | Adult_16 | FBtr0335025 | *osa#* | 3R |
| 108 | Adult_19 | FBtr0340106 | *Sec16* | X |
| 109 | Adult_20 | FBtr0331752 | *sgg** | X |
| 110 | Adult_21 | FBtr0100866 | *ATPase8* | Mitochondria |
| 111 | Adult_23 | FBtr0433498 | *ATPase6* | Mitochondria |
| 112 | Adult_24 | FBtr0331752 | *sgg** | X |
| 113 | Adult_25 | Fbtr0071091 | *CG1444* | X |
| 114 | Adult_31 | FBtr0087649 | *Lap7* | 2R |
| 115 | Adult_32 | FBtr0100866 | *ATPase8* | Mitochondria |
| 116 | Adult_33 | FBtr0331752 | *sgg** | X |
| 117 | Adult_35 | FBtr0344884 | *CR9284* | 2R |
| 118 | Adult_36 | FBtr0335273 | *drp12#* | 2R |
| 119 | Adult_39 | FBtr0433499 | *ND1* | Mitochondria |
| 120 | Adult_40 | FBtr0304964 | *Cam#* | 2R |
| 121 | Adult_41 | FBtr0333925 | *CG31324* | 3R |
| 122 | Adult_42 | FBtr0305691 | *jvl* | 3R |
| 123 | Adult_50 | No match | _ | _ |
| 124 | Adult_51 | FBtr0333352 | *Galt* | 2L |
| 125 | Adult_52 | FBtr0331752 | *sgg** | X |
| 126 | Adult_53 | No match | _ | _ |
| 127 | Adult_54 | FBtr0307900 | *Tomosyn* | X |
| 128 | Adult_56 | FBtr0330260 | *CG43737* | X |
| 129 | Adult_58 | FBtr0330260 | *CG43737* | X |
| 130 | Adult_59 | FBtr0305982 | *ATPsynCF6* | 3R |
| 131 | Adult_60 | Fbtr0333925 | *CG31324* | 3R |
| 132 | Adult_61 | FBtr0100866 | *ATPase8* | Mitochondria |
| 133 | Adult_62 | FBtr0089263 | *Dsp1* | X |
| 134 | Adult_63 | FBtr0304964 | *Cam* | 2R |
| 135 | Adult_80 | FBtr0086740 | *pAbp* | 2R |
| 136 | Adult_84 | FBtr0334027 | *CG5399* | 3R |
