# Supplementary figures and images for: Genomic and cDNA selection-amplification identifies transcriptome-wide binding sites for the Drosophila protein sex-lethal
Source: PLoS One. 2021 May 24;16(5):e0250592. doi: 10.1371/journal.pone.0250592 (PMC8143406; doi:10.1371/journal.pone.0250592)

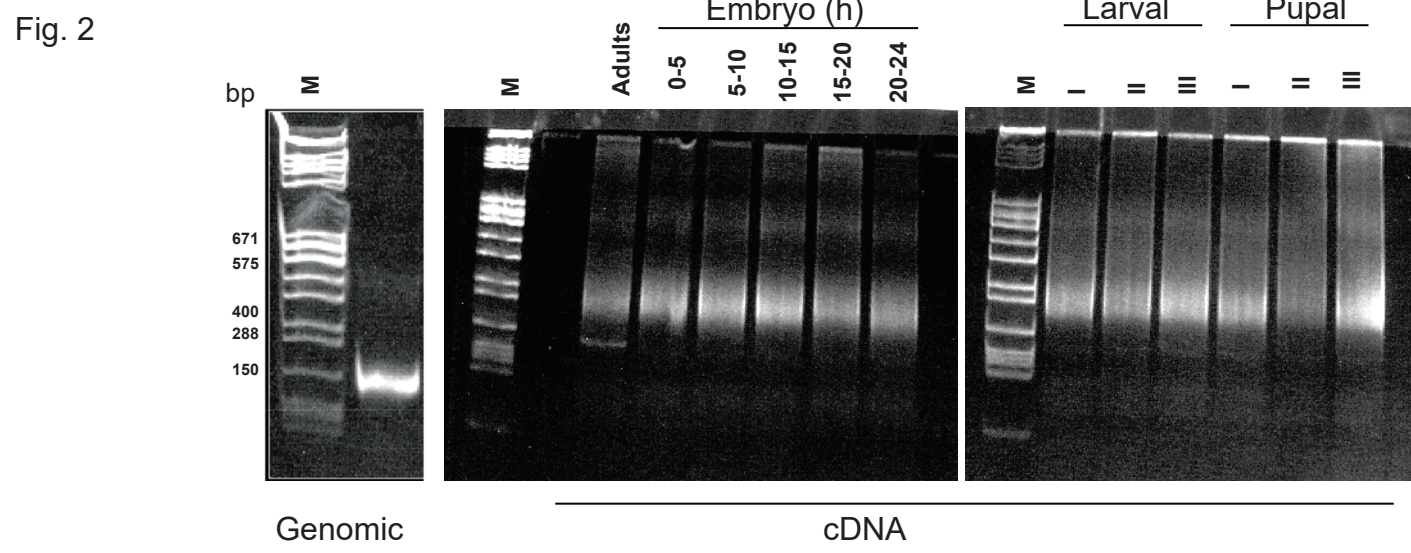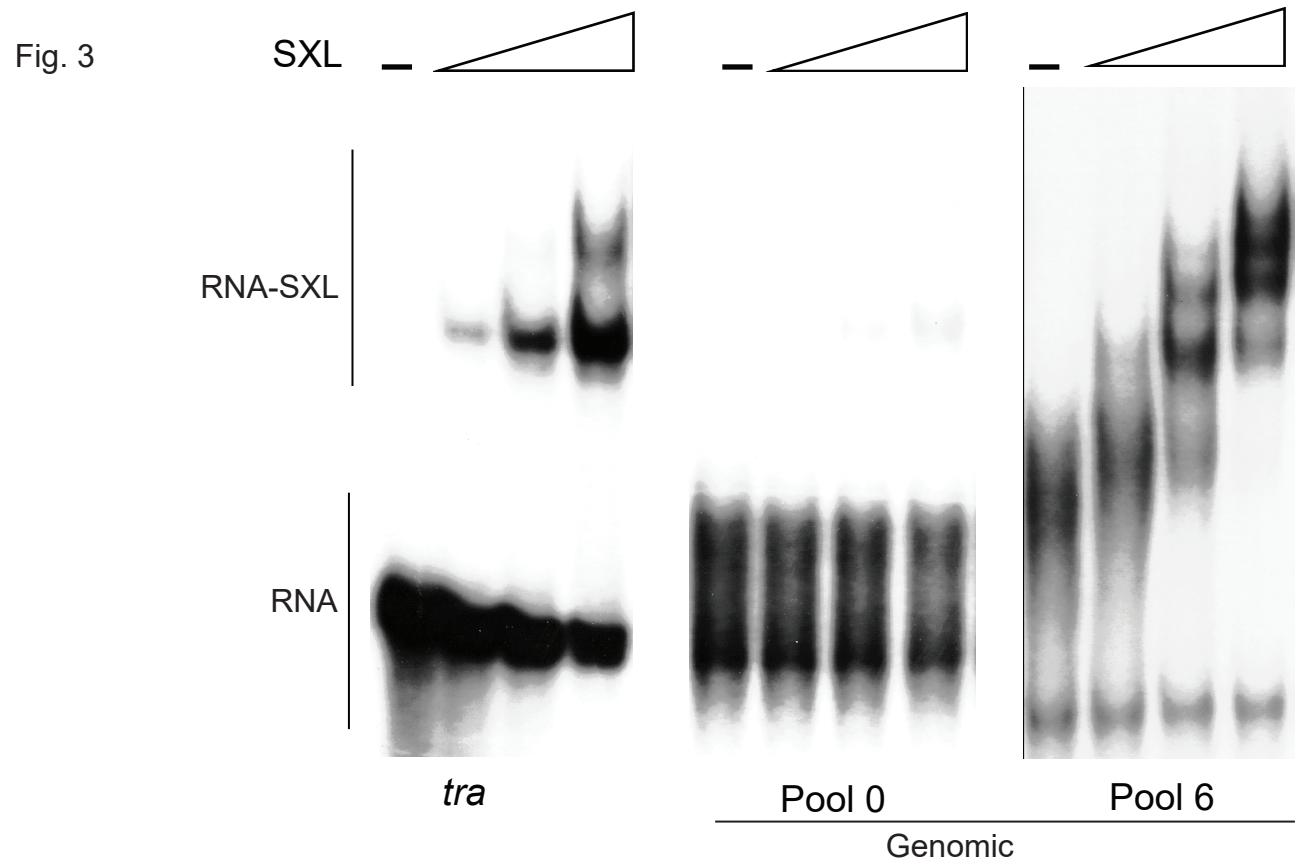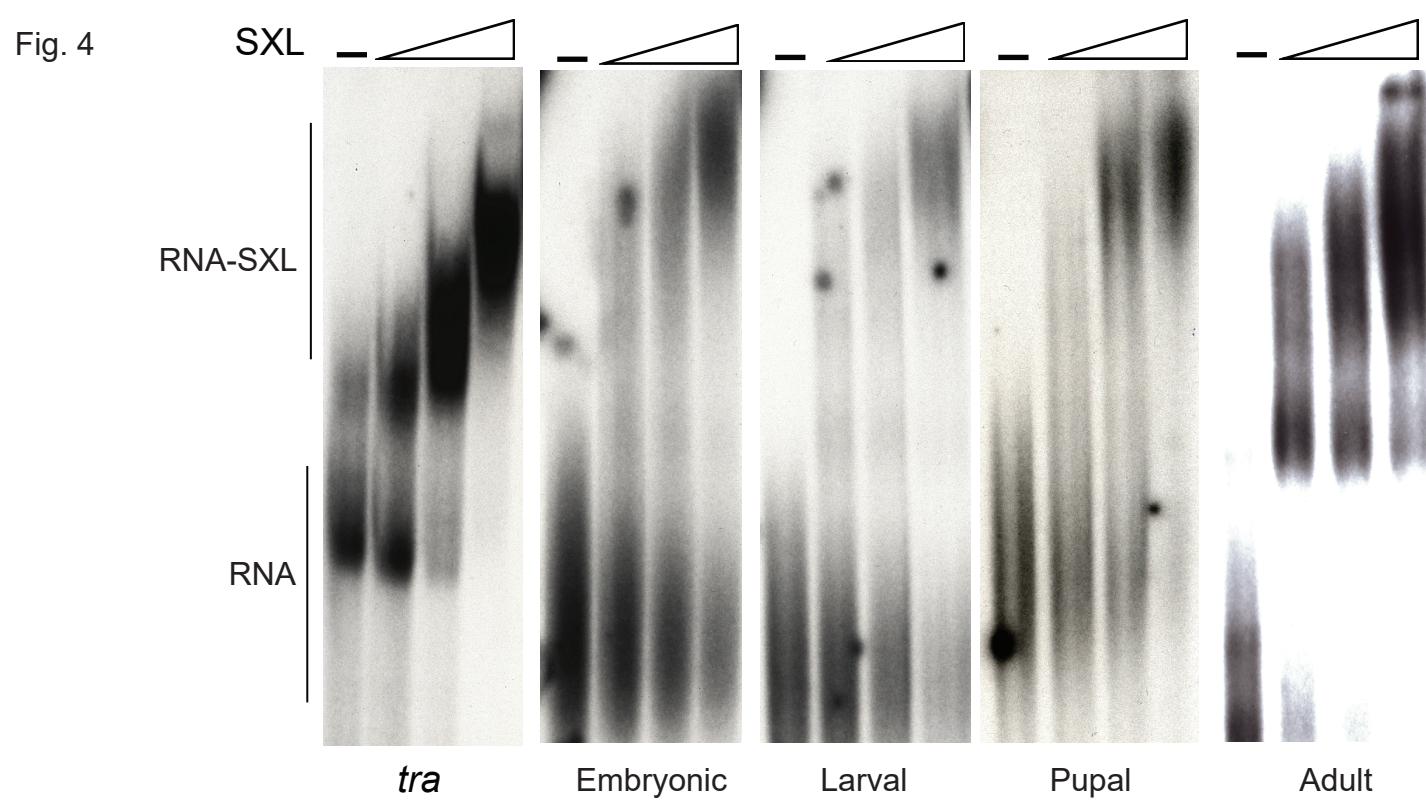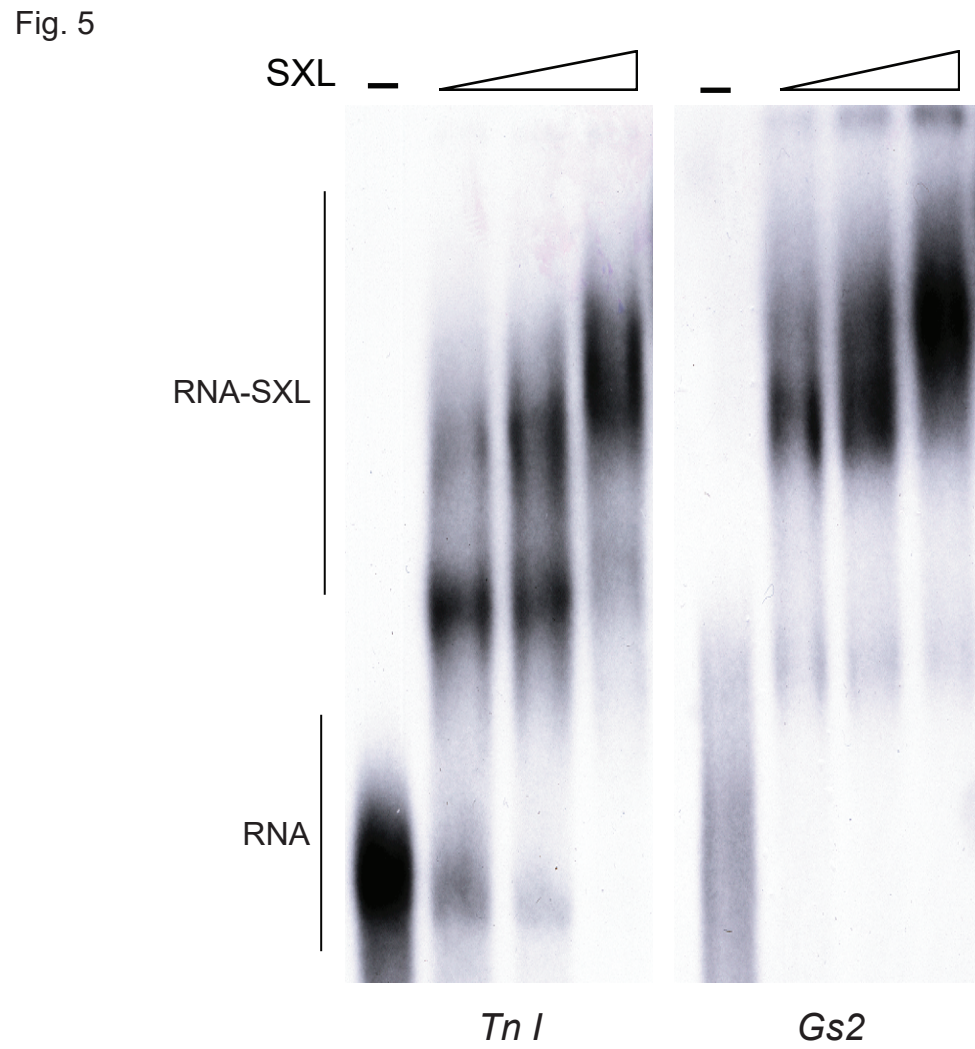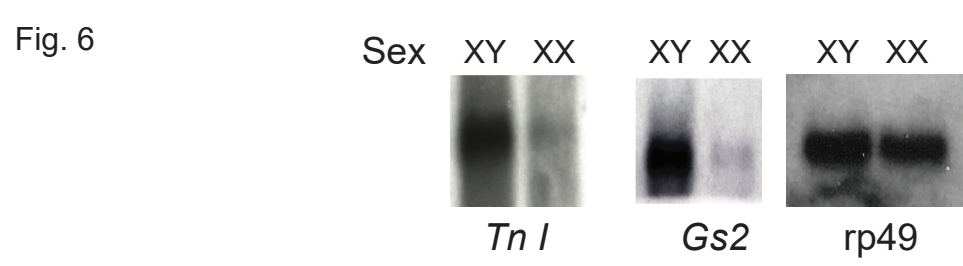

Supplement: S1 Raw images — (PDF) [file pone.0250592.s005.pdf]
